# The risk of all-cause and cause-specific mortality in people prescribed mirtazapine: an active comparator cohort study using electronic health records

**DOI:** 10.1186/s12916-022-02247-x

**Published:** 2022-02-02

**Authors:** Rebecca M. Joseph, Ruth H. Jack, Richard Morriss, Roger David Knaggs, Debbie Butler, Chris Hollis, Julia Hippisley-Cox, Carol Coupland

**Affiliations:** 1 Centre for Academic Primary Care, Lifespan and Population Health, School of Medicine, University of Nottingham, Nottingham, UK; 2 National Institute for Health Research MindTech MedTech Co-operative, The Institute of Mental Health, University of Nottingham, Nottingham, UK; 3 National Institute for Health Research Nottingham Biomedical Research Centre, Nottingham University Hospitals NHS Trust, Nottingham, UK; 4 Mental Health & Cognitive Neuroscience, School of Medicine, University of Nottingham, Nottingham, UK; 5 School of Pharmacy, University of Nottingham, Nottingham, UK; 6 Nuffield Department of Primary Care Health Sciences, University of Oxford, Oxford, UK

**Keywords:** Mirtazapine, Mortality, Depression, Antidepressants, Electronic health records

## Abstract

**Background:**

Studies have reported an increased risk of mortality among people prescribed mirtazapine compared to other antidepressants. The study aimed to compare all-cause and cause-specific mortality between adults prescribed mirtazapine or other second-line antidepressants.

**Methods:**

This cohort study used English primary care electronic medical records, hospital admission records, and mortality data from the Clinical Practice Research Datalink (CPRD), for the period 01 January 2005 to 30 November 2018. It included people aged 18–99 years with depression first prescribed a selective serotonin reuptake inhibitor (SSRI) and then prescribed mirtazapine (5081), a different SSRI (15,032), amitriptyline (3905), or venlafaxine (1580). Follow-up was from starting to stopping the second antidepressant, with a 6-month wash-out window, censoring at the end of CPRD follow-up or 30 November 2018. Age-sex standardised rates of all-cause mortality and death due to circulatory system disease, cancer, or respiratory system disease were calculated. Survival analyses were performed, accounting for baseline characteristics using inverse probability of treatment weighting.

**Results:**

The cohort contained 25,598 people (median age 41 years). The mirtazapine group had the highest standardised mortality rate, with an additional 7.8 (95% confidence interval (CI) 5.9–9.7) deaths/1000 person-years compared to the SSRI group. Within 2 years of follow-up, the risk of all-cause mortality was statistically significantly higher in the mirtazapine group than in the SSRI group (weighted hazard ratio (HR) 1.62, 95% CI 1.28–2.06). No significant difference was found between the mirtazapine group and the amitriptyline (HR 1.18, 95% CI 0.85–1.63) or venlafaxine (HR 1.11, 95% CI 0.60–2.05) groups. After 2 years, the risk was significantly higher in the mirtazapine group compared to the SSRI (HR 1.51, 95% CI 1.04–2.19), amitriptyline (HR 2.59, 95% CI 1.38–4.86), and venlafaxine (HR 2.35, 95% CI 1.02–5.44) groups. The risks of death due to cancer (HR 1.74, 95% CI 1.06–2.85) and respiratory system disease (HR 1.72, 95% CI 1.07–2.77) were significantly higher in the mirtazapine than in the SSRI group.

**Conclusions:**

Mortality was higher in people prescribed mirtazapine than people prescribed a second SSRI, possibly reflecting residual differences in other risk factors between the groups. Identifying these potential health risks when prescribing mirtazapine may help reduce the risk of mortality.

**Supplementary Information:**

The online version contains supplementary material available at 10.1186/s12916-022-02247-x.

## Background

Antidepressant prescribing rates are increasing over time in the United Kingdom (UK) [1] and elsewhere [2, 3], and mirtazapine is one of the ten most frequently prescribed antidepressants in several countries [3–5]. There is some evidence from UK-based studies of an increased mortality rate among people taking mirtazapine compared to other antidepressants [6–8]. However, it is likely that people prescribed mirtazapine differ in terms of characteristics like age, depression severity, and comorbidities compared to people prescribed selective serotonin reuptake inhibitors (SSRIs) or other antidepressants. Prescribers are also likely to take side effects and patient preference into account when prescribing antidepressants. Such factors could confound the relationship between mirtazapine and mortality.

To better understand the association between mirtazapine and mortality, we explored different causes of death while accounting for potential confounding factors. With the rationale that mirtazapine is not recommended as a first-line treatment in the UK [9, 10], the study groups were based on the second antidepressant prescribed. The aim of the study was therefore to compare the risks of all-cause and cause-specific mortality between adults with a diagnosis of depression who were prescribed mirtazapine, an SSRI, amitriptyline, or venlafaxine as second-line antidepressant treatments.

## Methods

The study protocol [11], code lists [12], and statistical code [13] are available online. The study used anonymised data provided under licence by the Clinical Practice Research Datalink (CPRD) [14, 15] and was reviewed and approved by the Independent Scientific Advisory Committee (reference 19_241).

### Data sources

The study used anonymised routinely collected electronic health records from general practices in England, UK (CPRD GOLD, November 2019), which capture all interactions between an individual and primary care and include coded information about demographic characteristics, lifestyle characteristics, diagnoses (coded using the Read v2 terminology), prescriptions, and more.

Primary care data were linked to Office for National Statistics (ONS) mortality data, Hospital Episode Statistics (HES)-admitted patient care data, and person-level deprivation measures (linkage set 17). Linkage is performed for CPRD by a trusted third party based on the National Health Service (NHS) number, date of birth, sex, and postcode. The ONS mortality data include the date and underlying cause of death, coded using ICD-10 codes.

### Study population

The study population was adults with a diagnosis of depression, initially prescribed an SSRI and subsequently prescribed mirtazapine, a different SSRI, amitriptyline, or venlafaxine. People were included if their general practice was linked to the HES and ONS datasets; their first recorded antidepressant was an SSRI and was prescribed between 01 January 2005 and 30 November 2018, when aged 18–99 years, and after at least 1 year of up-to-standard follow-up in CPRD; the second antidepressant was prescribed during or less than 90 days after the end of a course of the first SSRI (to capture treatment augmentation or switching rather than a new course of treatment); and they had a read code for depression on or before the first prescription date for the second antidepressant and less than 1 year before the first SSRI prescription. People who had a record of bipolar disorder or schizophrenia on or before starting the second antidepressant were excluded.

Follow-up was from starting the second antidepressant (index date) to the earliest of the end of the first course of the second antidepressant (see below), last data collection date, patient transfer out date, death, or 30 November 2018. Follow-up was censored if the person was prescribed a third antidepressant.

### Exposure

People were grouped according to whether their second antidepressant was mirtazapine, a different SSRI, amitriptyline, or venlafaxine. A published algorithm [16] was used to estimate a stop date for each antidepressant prescription, using recorded information such as daily dose and number of tablets prescribed (see Additional file 1: S1, Fig. S1). Drug exposure windows were considered continuous where the prescription start and stop dates overlapped and included a 6-month risk carry-over window after each prescription.

The daily dose was estimated by multiplying the daily number of tablets by the strength of the tablets. This was converted to the defined daily dose (DDD) using values from the World Health Organization searchable index [17]. For the sub-analysis investigating the effects of time-varying antidepressant dose, a time-varying current dose variable was created. Where prescriptions for the same antidepressant overlapped, doses were summed. Each resulting dosage period between the index date and follow-up end was included in the time-varying dose analysis.

### Outcomes

The date and underlying cause of death were extracted from the ONS mortality data. Cause of death was categorised using the ONS short list of cause of death [18]. Analyses of cause-specific mortality focused on deaths due to diseases of the circulatory system (ICD-10 codes I00–I99), deaths due to neoplasms (C00–D48), and deaths due to diseases of the respiratory system (J11–J99). These were the most frequent causes of death in the study cohort. An analysis exploring a combined self-harm/suicide outcome will be presented in a separate piece of work.

### Other variables

As described below, we used propensity score techniques to account for the differences in the person’s characteristics at the index date (baseline characteristics) between the four antidepressant exposure groups. The variables used to calculate the propensity scores were potential confounding factors and factors likely to be associated with mortality. These included factors in the Charlson Comorbidity Index [19, 20] and the QMortality risk prediction algorithm [21] and were defined using the primary care data. Where possible, we sourced code lists from the ClinicalCodes repository [22], the CALIBER phenotype resource [23], and individual papers.

Additional file 1: S2 contains the full list of variables and further details about how they were defined [22–34]. Demographic characteristics were age, sex, ethnicity, deprivation (quintile of Townsend Score [25]), and practice region. The most recent body mass index (BMI), smoking status, and alcohol use status on or prior to the index date were defined. A range of comorbidities was defined (see Additional file 1: S2 for the full list) and comorbidities were considered present if recorded on or before the index date. An indicator of a hospital record for intentional self-harm was also defined. Recent use of the following medicines was based on prescriptions recorded in the 6 months prior to the index date: opioids, glucocorticoids, non-steroidal anti-inflammatory drugs, other analgesics, statins, antipsychotics, anxiolytics, and hypnotic agents. An indicator of severe depression was defined as having a coded record of severe depression or depression with psychosis, scoring 15 or above on the Patient Health Questionnaire-9 (PHQ-9) scale, or scoring 16 or above on the Hospital Anxiety and Depression (HAD) scale. Other variables were the type of SSRI first prescribed and whether it was still being prescribed at the index date, the most recent and the current dose at the index date for the first SSRI prescribed, the time between starting the first and second antidepressants, and the calendar year of the index date.

### Analysis

The baseline characteristics of the four antidepressant groups were summarised. The most frequent causes of death in the study population were tabulated. Crude and age-sex standardised mortality rates were calculated using direct standardisation and the age-sex structure of the whole study population.

Survival analyses using Cox regression (all-cause mortality) or Fine-Gray regression [35] for competing risks (cause-specific mortality) were performed. The proportional hazards assumption was tested and where necessary interactions with calendar time were included in the models. Age-sex adjusted and propensity-score weighted models were performed, using stabilised inverse probability of treatment weights (IPTW) [36]. Propensity scores were estimated using multinomial logistic regression and included variables associated with the outcome (mortality) or with both the treatment group and the outcome, as recommended by Brookhart et al. [34]. To evaluate the propensity score models, we performed goodness-of-fit tests, assessed the overlap in propensities between the exposure groups graphically, and tested the balance of each variable after weighting using the analysis of variance (ANOVA) or chi-squared tests as appropriate. Further details, including the list of variables in the final model, are provided as supplementary files (Additional file 1: S3, Additional file 2: Table S1).

Multiple imputation by chained equations was used to estimate missing values of BMI, ethnicity, smoking status, alcohol use status, and deprivation. All variables used to estimate the propensity scores were included in the imputation models, as well as outcome and time to event variables. Twenty datasets were imputed. Propensity scores were estimated and weighted analyses performed on each of the twenty imputed datasets before combining the results using Rubin’s rules [37] (the ‘across imputation’ methodology described by Granger et al. [38]). A summary of missing data is provided in Additional file 3: Tables S2-S3.

All data handling and analyses were conducted using Stata MP/16.1. A significance level of 0.05 was used throughout. Small cell counts in tables have been masked.

### Sensitivity analyses

We repeated the main analysis including the current antidepressant dose as a time-varying covariate. We compared those in the mirtazapine group who ‘switched’ treatment with those who ‘augmented’ treatment, categorising people based on whether or not they had an active SSRI prescription 3 months after their first mirtazapine prescription (advancing the index date by three months).

We recalculated standardised mortality rates after excluding people with a record of palliative/end-of-life care or self-harm at the index date and including people with bipolar disorder or schizophrenia at the index date. Survival analyses were repeated restricting to ‘complete cases’ and excluding variables with missing data from the propensity score models. We also repeated the survival analyses: using all defined covariates to estimate propensity scores, using multivariable Cox regression instead of IPTW weighting, stratifying by age (18–64 and 65–99 years), adjusting the risk carry-over window (0 days, 30 days, and to follow-up end), applying maximum follow-up windows (1 year, 5 years), and excluding people with a record of cancer or self-harm at the index date.

### Patient and public involvement (PPI)

The study team included two PPI representatives who contributed to the discussions at all stages of the study, one of whom (DB) is a co-author of this paper. In addition, the project was discussed with the MindTech Involvement Team, a group with lived experience of mental health conditions.

## Results

**Fig. 1:**
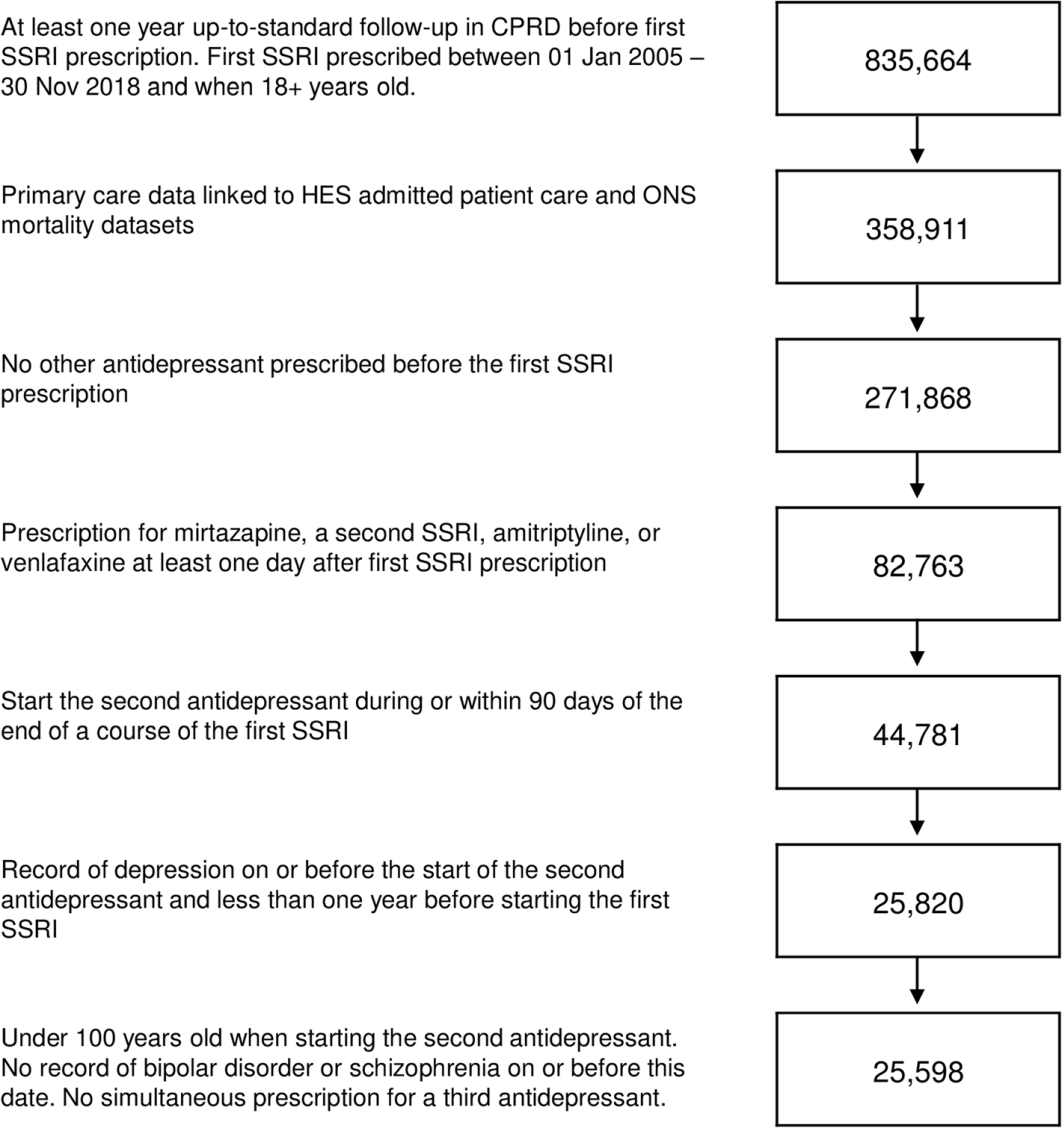
Flow diagram showing the definition of the study population. CPRD, Clinical Practice Research Datalink; SSRI, selective serotonin reuptake inhibitor; HES, hospital episode statistics; ONS, Office for National Statistics

Figure 1 shows the derivation of the study population. The final study population included 25,598 people, from 380 general practices, of whom 5081 were prescribed mirtazapine, 15,032 a different SSRI, 3905 amitriptyline, and 1580 venlafaxine.

Baseline demographic and lifestyle characteristics of the study population are summarised in Table 1. The full baseline characteristics are presented in Additional file 4: Table S4. Overall, 58.5% of the study population were female. This proportion differed between the exposure groups and was the lowest in the mirtazapine group (48.7% female). The mirtazapine group had the highest baseline rates of current smoking (36.0%), heavy drinking (6.7%), alcohol misuse (5.1%), and substance misuse disorder (4.1%). They also had the highest prevalence of self-harm (8.2%) and recent prescriptions for hypnotic agents (24.8%) at baseline.

**Table 1:** Demographic and lifestyle characteristics at index date, by study group

	All	Mirtazapine	SSRI	Amitriptyline	Venlafaxine
Count	25,598	5081	15,032	3905	1580
Age, median (IQR), years	41 (29–54)	43 (30–58)	38 (27–50)	48 (37–60)	40 (30–50)
Sex, no. (%)					
Male	10,617 (41.5%)	2609 (51.3%)	5937 (39.5%)	1346 (34.5%)	725 (45.9%)
Female	14,981 (58.5%)	2472 (48.7%)	9095 (60.5%)	2559 (65.5%)	855 (54.1%)
Ethnicity, no. (%)^a^					
Asian or Asian British	457 (2.4%)	110 (2.9%)	241 (2.2%)	83 (2.7%)	23 (2.1%)
Black or Black British	274 (1.5%)	41 (1.1%)	159 (1.5%)	57 (1.9%)	17 (1.5%)
Mixed	167 (0.9%)	38 (1.0%)	99 (0.9%)	19 (0.6%)	11 (1.0%)
Chinese or other ethnic groups	219 (1.2%)	35 (0.9%)	140 (1.3%)	32 (1.0%)	12 (1.1%)
White	17,692 (94.1%)	3622 (94.2%)	10,151 (94.1%)	2870 (93.8%)	1049 (94.3%)
Missing ethnicity, no. (%)	6789 (26.5%)	1235 (24.3%)	4242 (28.2%)	844 (21.6%)	468 (29.6%)
Deprivation score (Townsend quintile), no. (%)^a^					
1 (least deprived)	4977 (19.5%)	893 (17.6%)	2944 (19.6%)	806 (20.6%)	334 (21.2%)
2	5126 (20.0%)	982 (19.3%)	2996 (20.0%)	778 (19.9%)	370 (23.4%)
3	5514 (21.6%)	1066 (21.0%)	3241 (21.6%)	861 (22.0%)	346 (21.9%)
4	5587 (21.8%)	1129 (22.2%)	3343 (22.3%)	839 (21.5%)	276 (17.5%)
5 (most deprived)	4372 (17.1%)	1006 (19.8%)	2492 (16.6%)	621 (15.9%)	253 (16.0%)
Missing deprivation score, no. (%)	30 (0.1%)^b^	5 (0.1%)	16 (0.1%)	< 5	< 5
Practice region, no. (%)					
North East	530 (2.1%)	120 (2.4%)	289 (1.9%)	93 (2.4%)	28 (1.8%)
North West	4524 (17.7%)	1277 (25.1%)	2419 (16.1%)	592 (15.2%)	236 (14.9%)
Yorkshire and the Humber	780 (3.0%)	127 (2.5%)	493 (3.3%)	131 (3.4%)	29 (1.8%)
East Midlands	656 (2.6%)	94 (1.9%)	439 (2.9%)	105 (2.7%)	18 (1.1%)
West Midlands	3188 (12.5%)	525 (10.3%)	1963 (13.1%)	443 (11.3%)	257 (16.3%)
East of England	2286 (8.9%)	359 (7.1%)	1327 (8.8%)	384 (9.8%)	216 (13.7%)
South West	3572 (14.0%)	782 (15.4%)	1939 (12.9%)	608 (15.6%)	243 (15.4%)
South Central	3288 (12.8%)	562 (11.1%)	1957 (13.0%)	624 (16.0%)	145 (9.2%)
London	2628 (10.3%)	479 (9.4%)	1622 (10.8%)	381 (9.8%)	146 (9.2%)
South East Coast	4146 (16.2%)	756 (14.9%)	2584 (17.2%)	544 (13.9%)	262 (16.6%)
BMI, median (IQR)^a^	26.2 (22.8–30.7)	25.6 (22.4–29.7)	26.1 (22.7–30.5)	27.2 (23.5–32.1)	26.6 (23.1–31.1)
Missing BMI, no. (%)	7338 (28.7%)	1529 (30.1%)	4541 (30.2%)	818 (20.9%)	450 (28.5%)
Smoking status, no. (%)^a^					
Never	9941 (40.0%)	1854 (37.6%)	5903 (40.6%)	1525 (39.7%)	659 (43.2%)
Former	6762 (27.2%)	1300 (26.4%)	3853 (26.5%)	1197 (31.1%)	412 (27.0%)
Current	8146 (32.8%)	1771 (36.0%)	4797 (33.0%)	1124 (29.2%)	454 (29.8%)
Missing smoking status, no. (%)	749 (2.9%)	156 (3.1%)	479 (3.2%)	59 (1.5%)	55 (3.5%)
Alcohol intake, no. (%)^a^					
Non-drinker	3320 (33.3%)	647 (31.6%)	1945 (34.2%)	543 (32.0%)	185 (34.1%)
Former drinker	1438 (14.4%)	334 (16.3%)	764 (13.4%)	272 (16.0%)	68 (12.5%)
Occasional drinker	4226 (42.4%)	825 (40.3%)	2416 (42.5%)	753 (44.3%)	232 (42.8%)
Moderate drinker	467 (4.7%)	104 (5.1%)	264 (4.6%)	72 (4.2%)	27 (5.0%)
Heavy drinker	520 (5.2%)	138 (6.7%)	293 (5.2%)	59 (3.5%)	30 (5.5%)
Missing alcohol intake, no. (%)	15,627 (61.0%)	3033 (59.7%)	9350 (62.2%)	2206 (56.5%)	1038 (65.7%)

*SSRI* selective serotonin reuptake inhibitor, *IQR* interquartile range, *BMI* body mass index

^a^Not counting people with missing values

^b^Values rounded to mask small numbers

### All-cause mortality

Total follow-up was 37,209 person-years, and the median length of follow-up was 8 months (interquartile range 6.2–18.6 months). There were 599 deaths; thus, the overall crude mortality rate was 16.1 per 1000 person-years (95% confidence interval (CI) 14.9–17.4). The age-sex standardised mortality rate was higher in the mirtazapine group than in the other three groups, with an additional 7.8 (95% CI 5.9–9.7) deaths per 1000 person-years compared to the SSRI group (Table 2).

**Table 2:** Crude and age-sex standardised mortality rates for all-cause and cause-specific mortality

Group	Number of deaths	Person-years	Crude mortality rate/1000 pereson-years (95% CI)	Standardised mortality rate/1000 person-years (95% CI)	Excess risk/1000 person-years (95% CI)
**All-cause mortality**					
All	599	37,209	16.1 (14.9–17.4)	16.1 (14.8–17.4)	
Mirtazapine	213	6361	33.5 (29.3–38.3)	21.6 (18.5–25.0)	Reference
SSRI	251	23,224	10.8 (9.6–12.2)	13.8 (12.1–15.6)	− 7.8 (− 9.7 to − 5.9)
Amitriptyline	107	4744	22.6 (18.7–27.3)	17.6 (14.3–21.5)	− 4.0 (− 6.0 to − 2.0)
Venlafaxine	28	2880	9.7 (6.7–14.1)	18.9 (11.3–29.2)	− 2.6 (− 4.7 to − 0.6)
**Deaths due to diseases of the circulatory system**					
All	159	37,209	4.3 (3.7–5.0)	4.3 (3.6–5.0)	
Mirtazapine	51	6361	8.0 (6.1–10.5)	4.8 (3.5–6.5)	Reference
SSRI	73	23,224	3.1 (2.5–4.0)	4.0 (3.2–5.1)	− 0.7 (− 1.7 to 0.2)
Amitriptyline	28	4744	5.9 (4.1–8.5)	4.3 (2.8–6.4)	− 0.5 (− 1.4 to 0.5)
Venlafaxine	7	2880	2.4 (1.2–5.1)	7.0 (2.4–14.9)	2.2 (1.1 to 3.3)
**Deaths due to diseases of the respiratory system**					
All	106	37,209	2.8 (2.4–3.4)	2.8 (2.3–3.4)	
Mirtazapine	37	6361	5.8 (4.2–8.0)	3.6 (2.5–5.1)	Reference
SSRI	45	23,224	1.9 (1.4–2.6)	2.6 (1.9–3.4)	− 1.0 (− 1.8 to − 0.3)
Amitriptyline	17	4744	3.6 (2.2–5.8)	2.6 (1.5–4.4)	− 1.0 (− 1.8 to − 0.2)
Venlafaxine	7	2880	2.4 (1.2–5.1)	4.0 (1.2–9.5)	0.4 (− 0.5 to 1.3)
**Deaths due to neoplasms**					
All	156	37,209	4.2 (3.6–4.9)	4.2 (3.6–4.9)	
Mirtazapine	55	6361	8.6 (6.6–11.3)	6.0 (4.4–8.0)	Reference
SSRI	55	23,224	2.4 (1.8–3.1)	3.0 (2.2–3.9)	− 3.0 (− 3.9 to − 2.0)
Amitriptyline	38	4744	8.0 (5.8–11.0)	6.4 (4.4–8.9)	0.4 (− 0.7 to 1.6)
Venlafaxine	8	2880	2.8 (1.4–5.6)	3.4 (1.4–7.3)	− 2.6 (− 3.5 to − 1.6)

Standardised mortality rates are age-sex standardised using the structure of the overall study population

In the survival analyses, an interaction between the exposure group and time (less than/greater than 2 years after index date) was included in the model to meet the proportional hazards assumption (mortality rates for these two time periods are summarised in Additional file 5: Table S5). For follow-up of < 2 years, mortality was statistically significantly higher in the mirtazapine group compared to the SSRI group (hazard ratio (HR) 1.62, 95% CI 1.28–2.06). We found no significant difference in mortality between the mirtazapine group and the amitriptyline (HR 1.18, 95% CI 0.85–1.63) and venlafaxine groups (HR 1.11, 95% CI 0.60–2.05) (Table 3). For follow-up of 2+ years, mortality was significantly higher in the mirtazapine group compared to each of the other groups in the IPTW-weighted models.

**Table 3:** Survival analyses comparing the risk of all-cause and cause-specific mortality between the study groups

	Unadjusted	Age-sex adjusted	Propensity score-weighted
**All-cause mortality, hazard ratios (95% CI)**^**a**^			
*Time < 2 years from starting treatment*			
Mirtazapine/SSRI	3.19 (2.56–3.97)	1.58 (1.27–1.97)	1.62 (1.28–2.06)
Mirtazapine/amitriptyline	1.35 (1.04–1.75)	1.12 (0.86–1.45)	1.18 (0.85–1.63)
Mirtazapine/venlafaxine	3.10 (1.92–4.99)	1.26 (0.78–2.03)	1.11 (0.60–2.05)
*Time 2+ years from starting treatment*			
Mirtazapine/SSRI	2.68 (1.92–3.75)	1.26 (0.90–1.76)	1.51 (1.04–2.19)
Mirtazapine/amitriptyline	2.28 (1.34–3.87)	2.01 (1.18–3.41)	2.59 (1.38–4.86)
Mirtazapine/venlafaxine	3.64 (1.80–7.35)	1.33 (0.66–2.70)	2.35 (1.02–5.44)
**Deaths due to diseases of the circulatory system, subdistribution hazard ratios (95% CI)**			
Mirtazapine/SSRI	2.42 (1.70–3.46)	1.10 (0.76–1.58)	1.41 (0.96–2.08)
Mirtazapine/amitriptyline	1.35 (0.85–2.16)	1.04 (0.64–1.67)	1.11 (0.65–1.88)
Mirtazapine/venlafaxine	3.07 (1.39–6.77)	1.09 (0.49–2.43)	0.74 (0.27–2.01)
**Deaths due to diseases of the respiratory system, subdistribution hazard ratios (95% CI)**			
Mirtazapine/SSRI	2.86 (1.86–4.42)	1.24 (0.80–1.94)	1.72 (1.07–2.77)
Mirtazapine/amitriptyline	1.60 (0.90–2.84)	1.21 (0.68–2.14)	1.40 (0.73–2.68)
Mirtazapine/venlafaxine	2.24 (1.00–5.03)	0.73 (0.34–1.55)	1.53 (0.62–3.75)
**Deaths due to neoplasms, subdistribution hazard ratios (95% CI)**^**a**^			
*Time < 2 years from starting treatment*			
Mirtazapine/SSRI	3.82 (2.44–5.98)	2.08 (1.32–3.29)	1.74 (1.06–2.85)
Mirtazapine/amitriptyline	0.86 (0.55–1.34)	0.74 (0.47–1.16)	1.08 (0.64–1.81)
Mirtazapine/venlafaxine	2.46 (1.04–5.78)	1.19 (0.50–2.84)	1.14 (0.42–3.09)
*Time 2+ years from starting treatment*			
Mirtazapine/SSRI	2.82 (1.42–5.60)	1.44 (0.72–2.88)	1.86 (0.89–3.89)
Mirtazapine/amitriptyline	9.90 (1.29–75.84)	8.21 (1.06–63.35)	9.37 (1.20–73.29)
Mirtazapine/venlafaxine	3.94 (0.90–17.36)	1.74 (0.39–7.78)	4.79 (1.00–22.99)

Cox regression was performed for all-cause mortality and Fine-Gray (competing risk) regression for cause-specific mortality

*CI* confidence interval, *SSRI* selective serotonin reuptake inhibitor

^a^Model includes interaction between the study group and follow-up time, split at time = 2 years

### Cause-specific mortality

The most frequent causes of death (those with at least 10 recorded deaths) are summarised in Table 4. Diseases of the circulatory system (159 deaths), diseases of the respiratory system (106 deaths), and neoplasms (156 deaths) accounted for 70% of all deaths in the study population. Standardised rates of death due to circulatory system diseases were similar between groups, with a slightly higher rate in the venlafaxine group (2.2 additional deaths per 1000 person-years compared to the mirtazapine group) (Table 2). There was little difference in the rates of death due to respiratory system diseases between the groups. For deaths due to neoplasms, standardised rates were higher in the mirtazapine group compared to the SSRI and venlafaxine groups (approximately 3 additional deaths per 1000 person-years).

**Table 4:** Most common causes of death during follow-up

ICD-10 codes	Chapter/subheading	Number of deaths				
		Mirtazapine	SSRI	Amitriptyline	Venlafaxine	Total
	**All deaths**	**213**	**251**	**107**	**28**	**599**
**I00–I99**	**IX. Diseases of the circulatory system**	**50**	**70**	**25**	**5**	**159**
I20–I25	Ischaemic heart diseases	15	35	15	< 5	69
I60–I69	Cerebrovascular diseases	15	15	5	5	42
I26–I51	Other heart diseases	15	10	< 5	< 5	29
I21–I22	Acute myocardial infarction	< 5	10	< 5	< 5	21
I64	Stroke, not specified as haemorrhage or infarction	5	5	< 5	< 5	20
**C00–D48**	**II. Neoplasms**	**55**	**55**	**35**	**5**	**156**
C33–C34	Malignant neoplasm of the trachea, bronchus, and lung	5	10	10	< 5	34
C61	Malignant neoplasm of prostate	5	< 5	5	< 5	15
C15	Malignant neoplasm of oesophagus	5	5	< 5	< 5	12
**J00–J99**	**X. Diseases of the respiratory system**	**35**	**45**	**15**	**5**	**106**
J40–J44	Bronchitis, emphysema, and other chronic obstructive pulmonary diseases	10	20	5	< 5	42
J12–J18	Pneumonia	10	10	5	< 5	38
**V01–Y89; U50.9**	**XX. External causes of morbidity and mortality**	**15**	**15**	**5**	**< 5**	**40**
X60–X84; Y10–Y341	Intentional self-harm and event of undetermined intent	10	10	< 5	< 5	22
**K00–K93**	**XI. Diseases of the digestive system**	**10**	**15**	**< 5**	**< 5**	**36**
K70–K77	Diseases of the liver	< 5	5	< 5	< 5	15
**F00–F99**	**V. Mental and behavioural disorders**	**10**	**10**	**< 5**	**< 5**	**27**
**G00–G99**	**VI. Diseases of the nervous system**	**5**	**10**	**5**	**< 5**	**24**
**R00–R99**	**XVIII. Symptoms, signs, and abnormal clinical and laboratory findings, not elsewhere classified**	**5**	**5**	**< 5**	**< 5**	**13**

Causes of death as classified in the ONS Short List Cause of Death [18]. Except for the first row and last column, numbers have been rounded to mask small cell counts

*ICD-10* International Classification of Diseases 10th Revision, *SSRI* selective serotonin reuptake inhibitor

In survival analyses, the risk of death due to diseases of the circulatory system did not differ significantly between the groups in the age-sex and IPTW-weighted models (Table 3). The risk of death due to diseases of the respiratory system was statistically significantly higher in the mirtazapine group compared to the SSRI group in the IPTW-weighted model (HR 1.72, 95% CI 1.07–2.77). For deaths due to neoplasms, an interaction between the exposure group and time (less than/greater than 2 years) was included in the models (Additional file 5: Table S6 shows rates broken down by time period). The risk was higher in the mirtazapine group than in the SSRI group in both time intervals, although the results were not statistically significant for follow-up of 2+ years. There were few events after 2 years in the amitriptyline and venlafaxine groups.

### Sensitivity analyses

Including current antidepressant dose in the Cox regression models for all-cause mortality had little impact on the comparisons between the exposure groups (Additional file 6: Table S7). Only the current amitriptyline dose was associated with mortality. We found no difference in the risk of all-cause mortality between those prescribed mirtazapine who stopped the original SSRI and those who continued the original SSRI (Additional file 7: Table S8).

Altering the inclusion criteria made little difference to all-cause mortality rates (Additional file 5: Table S9). For all-cause mortality, the results of multivariable-adjusted Cox regression were similar to those of the IPTW-weighted models for follow-up < 2 years (Additional file 8: Table S10). For follow-up of 2+ years, the risk differences between the mirtazapine group and the SSRI and venlafaxine groups were smaller and non-significant in the multivariable-adjusted model. The results of the other sensitivity analyses were similar to the main analysis (Additional file 8: Table S10).

For cause-specific mortality, using Cox regression instead of Fine-Gray regression produced similar results to the main analyses (Additional file 8: Tables S11-S13). The analyses of deaths due to neoplasms were repeated after excluding people with a record of cancer prior to the index date. Over half the deaths (90/156, 58%) were in people with a prior cancer record. The results were similar after excluding these people, although the comparison between the mirtazapine and SSRI groups was no longer statistically significant (Additional file 8: Table S13).

The results of the survival analyses restricting to complete cases (*n* = 6794) are shown in Additional file 8: Tables S10-S13. All results were non-significant in the complete case analyses, and the magnitude of some hazard ratios was altered. However, the sample size was much smaller.

## Discussion

People prescribed mirtazapine had a higher age-sex standardised all-cause mortality rate than people prescribed an SSRI, amitriptyline, or venlafaxine as a second-line antidepressant. There were differences in baseline characteristics between the mirtazapine group and the other groups, including increased rates of current smoking and heavy drinking. After accounting for baseline characteristics, the risks of all-cause mortality, death due to neoplasms, and death due to diseases of the respiratory system were statistically significantly higher in people prescribed mirtazapine compared to people prescribed an SSRI. For all-cause mortality and deaths due to neoplasms, these results were seen in the first 2 years of follow-up. After 2 years of follow-up, the risks of all-cause mortality and death due to neoplasms appeared to be increased for people prescribed mirtazapine compared to each of the other groups.

The results build on the existing evidence for an increased risk of mortality among people prescribed mirtazapine compared to those prescribed SSRIs. A study using the UK-based primary care database QResearch reported a 67% increased risk of all-cause mortality among mirtazapine users aged 20–64 years compared to citalopram users, after accounting for the baseline characteristics [7]. For adults aged 65 years or over, another QResearch study found mirtazapine and trazodone were associated with the highest hazard ratios for all-cause mortality among the 11 most commonly prescribed antidepressant drugs [6], and mirtazapine users had a 51% increased mortality risk compared to SSRI users in a study using a London-based secondary care database [8]. However, a large study using a German health insurance database (the German Pharmacoepidemiological Research Database, GePaRD) found a slightly lower risk of mortality in people aged 65 years or over prescribed mirtazapine compared to those prescribed citalopram (adjusted HR 0.94, 95% CI 0.92–0.97) which was attenuated after high-dimensional propensity score adjustment (HR 0.98, 95% CI 0.95–1.02) [39]. These contradictory results could relate to the differences in the study populations, the confounders accounted for, or how the antidepressants were compared. Unlike the other studies, Kollhorst et al. [39] restricted the comparison groups to new users, and they compared the first antidepressant treatment only.

### Strengths and limitations

The study was designed to reduce residual confounding between the study groups and is the first to focus on second-line mirtazapine use. The ‘new user’ design (with respect to the treatments compared) increases the likelihood that the people in the study were at a similar point in the course of their illness and treatment history at cohort entry [40, 41]. Comparing the active treatment groups rather than antidepressant users and non-users should also help to reduce the baseline differences as people who are prescribed antidepressants are likely to differ in terms of depression severity and comorbidities from those who are not [40, 41]. Finally, we used propensity score weighting to account for the baseline characteristics.

To reduce the risk of indication bias, we restricted the study population to people with a recorded diagnosis of depression. This, with the other cohort restrictions, reduced the sample size and generalisability of the study. However, the restrictions were applied to improve the validity of the results [40]. The dataset used is broadly representative of the UK population [14], and the study population is likely to represent typical patients prescribed mirtazapine, amitriptyline, venlafaxine, or a second SSRI to treat depression following the initial inadequate response to treatment with an SSRI.

The results differed somewhat when a complete case approach was used. The complete case analysis had a much smaller sample size (6794 compared to 25,598), which likely accounts for the comparisons in the complete case analysis being non-significant. The difference in the point estimates could suggest that the main analysis (using multiple imputation) was not fully adjusted for variables with missing data. When these variables were excluded from the analyses, the results were very similar to the main analyses using multiple imputation. However, it is also likely that people with complete data are systematically different to the whole study population so the complete case results should not be considered reflective of the study population.

In addition, the nature of observational research and electronic health records means residual and unmeasured confounding remain a possibility. Key factors, such as detailed information about mental health conditions, may not be routinely captured in read-coded primary care data and variables derived from such data may include a degree of misclassification. Misclassification is also likely through using prescription data as a proxy of drug use, as there is no guarantee that people took the medicine. In addition, information about prescribing in secondary care is not available in the datasets used. Thus, exposure history may have been incomplete, particularly for those with severe or difficult to treat depression who are more likely to be referred to specialists. These limitations may introduce a degree of bias, the direction of which is difficult to predict. Furthermore, some of the drug and outcome groups included small numbers resulting in reduced power and wide confidence intervals. Numerous comparisons were made, and statistical significance should not be over-emphasised.

## Conclusions

Our results suggest an increased risk of mortality associated with being prescribed mirtazapine compared to being prescribed an SSRI as a second-line antidepressant. However, this does not appear to be strongly driven by a particular cause of death. This finding, and the risk of residual confounding, means that we cannot provide evidence for a causal link between mirtazapine and mortality. It is perhaps more likely that residual differences between people prescribed mirtazapine rather than an SSRI account for the difference in risk of mortality. Ultimately, whether the increase in risk was related to individual factors or to the drug itself, the rate of mortality was highest in the mirtazapine group. Thus, people who are prescribed mirtazapine may need additional support to identify health risks and improve outcomes.

## Supplementary Information

**Additional file 1: Supplementary methods. S1.** Drug exposure windows. **Fig. S1.** Drug data preparation algorithm, with options used highlighted. **S2.** Other variables. **S3.** Propensity score model.

**Additional file 2: Table S1.** Propensity score model: multinomial logistic regression with mirtazapine as the reference group.

**Additional file 3: Table S2.** Proportion of people with complete data and proportion of deaths recorded in people with complete data. **Table S3.** Missingness for each variable by study group.

**Additional file 4: Table S4.** Full baseline characteristics.

**Additional file 5.** Sensitivity analyses: rates of all-cause mortality and mortality due to neoplasms. **Table S5.** Rates of all-cause mortality per 1000 person-years according to period of follow-up. **Table S6.** Sensitivity analysis – Rates of death due to neoplasms per 1000 person-years according to period of follow-up. Table S9. Sensitivity analysis – Rates of all-cause mortality per 1000 person-years after altering study inclusion criteria.

**Additional file 6: Table S7.** Cox regression, risk of all-cause mortality after accounting for current antidepressant dose.

**Additional file 7: Table S8.** Cox regression, risk of all-cause mortality according to switching to or augmenting SSRI with mirtazapine.

**Additional file 8.** Sensitivity analyses for all outcomes: varying the survival analysis models. **Table S10.** Sensitivity analyses for all-cause mortality: varying the survival analysis models. **Table S11.** Sensitivity analyses for deaths due to diseases of the circulatory system: varying the survival analysis models. **Table S12.** Sensitivity analyses for deaths due to diseases of the respiratory system: varying the survival analysis models. **Table S13.** Sensitivity analyses for deaths due to neoplasms: varying the survival analysis models.

## Data Availability

This study used anonymised electronic health records provided under licence by the Clinical Practice Research Datalink (CPRD) (www.cprd.com), and the data cannot be shared. Access to CPRD data is subject to protocol approval through the CPRD’s Research Data Governance process (formerly the Independent Scientific Advisory Committee) – for information, contact the RDG Secretariat at rdg@cprd.com. All code lists, statistical code used to prepare and analyse the data (in the form of Stata do-files), and other information required to replicate the analysis are available on Zenodo.org (10.5281/zenodo.4779024).
