# Supplementary material for: The risk of all-cause and cause-specific mortality in people prescribed mirtazapine: an active comparator cohort study using electronic health records
Source: BMC Med. 2022 Feb 2;20:43. doi: 10.1186/s12916-022-02247-x (PMC8809032; doi:10.1186/s12916-022-02247-x)
Supplement: Supplementary file 1 — Additional file 1: Supplementary methods. S1. Drug exposure windows. Fig. S1. Drug data preparation algorithm, with options used highlighted. S2. Other variables. S3. Propensity score model. [file 12916_2022_2247_MOESM1_ESM.docx]

Additional file 1 – Supplementary methods

S1. Drug exposure windows.

The start and stop dates of individual antidepressant prescriptions were defined using a published algorithm[16]. The start dates are typically the prescription date unless they have been adjusted for any overlapping time. Prescription duration is not entered for most prescriptions and must be estimated from other information. The algorithm used includes 10 decision steps, which are shown below with the decisions used highlighted.

Figure modified from Pye S et al 2018[16], which is available through a [Creative Commons CC BY](https://creativecommons.org/licenses/) licence.

**Fig. S1 Drug data preparation algorithm, with options used highlighted.**


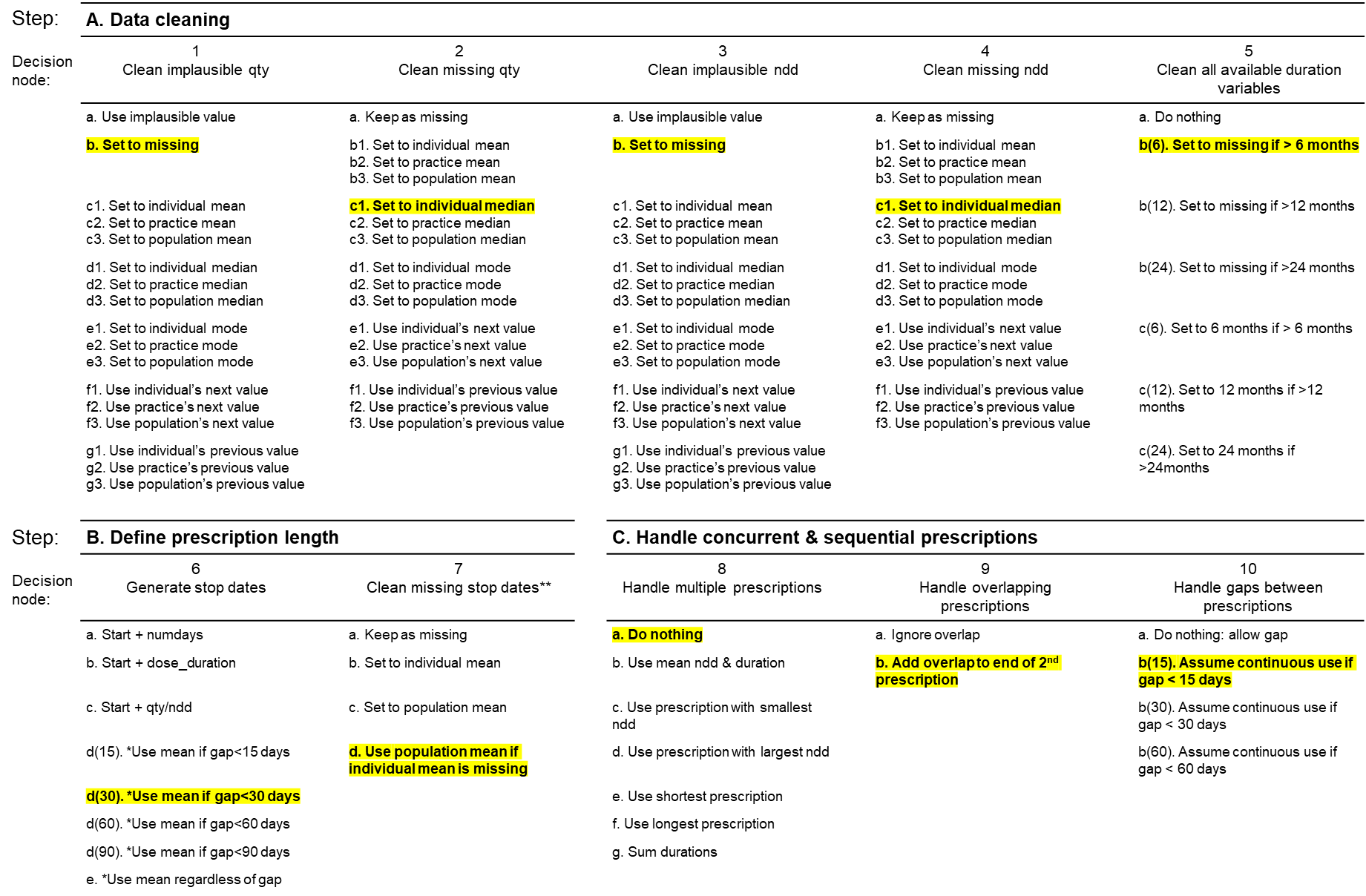


qty = total quantity entered by GP for the prescribed product; ndd = derived numeric daily dose; numdays = number of treatment days; dose_duration = derived duration of prescription. All options that produce a missing value stay coded as missing unless otherwise stated. *For options 6d-e: If only one is stop available, use it; if two are available and equal, use that date; if two available and unequal (but within X days), use mean; if three are available and unequal, use mean of closest two if within X days. **Records with missing stop dates after step 7 are dropped.

S2. Other variables.

Code lists

Read code lists and drug lists are available on Zenodo (<https://doi.org/10.5281/zenodo.4779024>) and the ClinicalCodes repository (<https://clinicalcodes.rss.mhs.man.ac.uk>). New code lists were developed if a list was not available in the ClinicalCodes repository[22], the CALIBER phenotyping resource[23], or individual papers. To develop a new list, an initial list of terms was extracted based on key word searches and then screened by clinicians in the research team.

Demographic and lifestyle characteristics

Age in index year (based on year of birth), sex, ethnicity (using the England and Wales 2001 Census broad ethnicity categories – Asian or Asian British, Black or Black British, Mixed, Chinese or other ethnic group, White – most recent code prior to index date)[24], socio-economic status (SES, quintile of Townsend Score[25]), and practice region. Ethnicity was defined using Read-coded information in the primary care record, supplemented with available secondary care information if missing. Body mass index (BMI) was defined using recorded weight and height measurements. The most recent BMI, smoking status (never/ former/ current)[26], and alcohol use status (non-/ former/ occasional/ moderate/ heavy drinker)[27] on or prior to index date were defined.

Prescription medicine

Use of the following drugs were considered present if prescribed on or less than 6 months before index date: opioids, glucocorticoids, non-steroidal anti-inflammatory drugs (NSAIDs), other analgesics, statins, antipsychotics, anxiolytics, hypnotic agents.

Comorbidities

The following comorbidities were considered present if recorded in the primary care data on or before the index date: abdominal pain, inflammatory bowel disease, indigestion, liver disease, obesity, pancreatitis, peptic ulcer disease, renal disease, anaemia, atrial fibrillation, angina, cerebrovascular disease, congestive heart failure, diabetes mellitus, hypertension, myocardial infarction[28], peripheral vascular disease, venous thromboembolism, alcohol misuse, anxiety[29], contact with mental health services, eating disorder, insomnia[30], intellectual disability, personality disorder, self-harm, substance misuse, appetite loss, living in a care home, hemiplegia, leg ulcer, palliative/end-of-life care, poor mobility, unexplained/unintentional weight loss, unplanned hospital admission, asthma, chronic obstructive pulmonary disease, dyspnoea[31], sleep apnoea, acquired immunodeficiency syndrome (AIDS), cancer, recent cancer (past year), metastatic tumour, dementia, epilepsy[32], fibromyalgia and generalised pain, Huntington's disease[33], migraine, multiple sclerosis, neuropathic pain, Parkinson's disease, and rheumatological disease. An indicator of a hospital record for intentional self-harm was also defined.

Other variables

Depression severity was defined as having a coded record of severe depression or depression with psychosis, scoring 15 or above on the Patient Health Questionnaire-9 (PHQ-9) scale, or scoring 16 or above on the Hospital Anxiety and Depression (HAD) scale. An indicator of having a record of completing a depression scale was defined. Other variables were: most recent dose of the first antidepressant on/prior to index date, current dose of first antidepressant at index date (including 0), whether or not the first antidepressant was still being prescribed at index date, the time between starting the first and second antidepressants, the first SSRI prescribed, and the calendar year of index date.

Time-varying antidepressant dose

Where prescriptions for the same antidepressant overlapped, doses were summed. Continuous periods of exposure on that drug with the same dosage were then collapsed. Each resulting dosage period between index date and end of follow-up was included in the time-varying dose analysis.

S3. Propensity score model.

To determine which covariates were used to estimate the propensity scores, the associations between each covariate and mortality were assessed using Cox regression. Those covariates association with mortality at the 0.05 level in univariate or age-sex adjusted models were used to estimate propensity scores. Thus the propensity score models included possible confounding variables and variables associated only with the outcome (mortality), but not variables associated only with treatment group, as recommended by Brookhart et al[34]. The propensity scores were then estimated using multinomial logistic regression. To evaluate propensity score models, goodness-of-fit tests were performed, overlap in propensities between exposure groups was determined graphically, and balance of each covariate after weighting was tested using ANOVA or Chi-squared tests as appropriate. Where necessary, transformed covariates and interactions were included in the models.

Variables included in the main propensity score model:

Sex, age, age^2^, age*sex, most recent antidepressant dose, 1/(most recent antidepressant dose), index year, self-harm (secondary care), self-harm (primary care), first antidepressant, antipsychotics, anxiolytics, glucocorticoids, hypnotics, opioids, statins, analgesics, severe depression, recent cancer, unexplained weight loss, venous thromboembolism, substance misuse, rheumatological disorders, renal failure, peripheral vascular disease, peptic ulcer disease, Parkinson’s disease, pancreatitis, palliative/end-of-life care, neuropathic pain, restricted mobility, migraine, myocardial infarction, metastatic tumour, leg ulcer, insomnia, indigestion, hypertension, unexpected hospital admission, hemiplegia, epilepsy, dyspnoea, diabetes with complications, diabetes, recorded depression scale, dementia, chronic obstructive pulmonary disorder, congestive heart failure, cerebrovascular disease, living in a care home, cancer, asthma, anxiety, appetite loss, angina, anaemia, alcohol misuse, atrial fibrillation. Variables containing imputed data: body mass index, deprivation score (Quintile of Townsend score), smoking status, alcohol intake, ethnicity.

Sensitivity analyses including all defined variables were also performed. Some variables were excluded as the models were not converging. Excluded variables were: AIDs, moderate liver disease, and Huntington’s disease.

**References** (see main article)
