# Supplementary material for: The risk of all-cause and cause-specific mortality in people prescribed mirtazapine: an active comparator cohort study using electronic health records
Source: BMC Med. 2022 Feb 2;20:43. doi: 10.1186/s12916-022-02247-x (PMC8809032; doi:10.1186/s12916-022-02247-x)
Supplement: Supplementary file 2 — Additional file 2: Table S1. Propensity score model: multinomial logistic regression with mirtazapine as the reference group. [file 12916_2022_2247_MOESM2_ESM.docx]

Additional file 2

**Table S1. Propensity score model: multinomial logistic regression with mirtazapine as the reference group.**

This is an example of one of the results tables out of the 20 imputed datasets (results were combined at a later analysis stage).

|  | **SSRI** | | **Amitriptyline** | | **Venlafaxine** | |
| --- | --- | --- | --- | --- | --- | --- |
|  | **Relative risk ratio (95% CI)** | **p-value** | **Relative risk ratio (95% CI)** | **p-value** | **Relative risk ratio (95% CI)** | **p-value** |
| Body mass index ^a^ | 1.01 (1.01-1.02) | <0.001 | 1.02 (1.01-1.03) | <0.001 | 1.02 (1.01-1.03) | <0.001 |
| Deprivation score (vs. 1, least deprived) ^a^ |  |  |  |  |  |  |
| 2 | 0.93 (0.83-1.03) | 0.165 | 0.83 (0.72-0.96) | 0.009 | 1.03 (0.86-1.23) | 0.750 |
| 3 | 0.88 (0.79-0.98) | 0.019 | 0.85 (0.74-0.97) | 0.019 | 0.83 (0.7-1) | 0.046 |
| 4 | 0.84 (0.75-0.93) | 0.001 | 0.77 (0.67-0.88) | <0.001 | 0.61 (0.5-0.73) | <0.001 |
| 5 (most deprived) | 0.69 (0.62-0.78) | <0.001 | 0.63 (0.54-0.73) | <0.001 | 0.65 (0.54-0.79) | <0.001 |
| Smoking status (vs. never smoker) ^a^ |  |  |  |  |  |  |
| Former smoker | 1.08 (0.99-1.18) | 0.077 | 1.03 (0.92-1.15) | 0.611 | 1.03 (0.89-1.2) | 0.668 |
| Current smoker | 0.89 (0.82-0.97) | 0.006 | 0.89 (0.8-1) | 0.048 | 0.76 (0.66-0.87) | <0.001 |
| Alcohol intake (vs. non-drinker) ^a^ |  |  |  |  |  |  |
| Former drinker | 0.92 (0.82-1.02) | 0.098 | 0.88 (0.77-1.02) | 0.082 | 1.02 (0.85-1.23) | 0.836 |
| Occasional drinker | 0.89 (0.83-0.97) | 0.005 | 0.87 (0.79-0.97) | 0.010 | 1.01 (0.88-1.16) | 0.908 |
| Moderate drinker | 0.83 (0.71-0.98) | 0.028 | 0.81 (0.65-1.02) | 0.068 | 1.05 (0.79-1.39) | 0.725 |
| Heavy drinker | 0.81 (0.69-0.96) | 0.017 | 0.79 (0.63-1) | 0.051 | 1.31 (0.99-1.74) | 0.056 |
| Ethnicity (vs. White) ^a^ |  |  |  |  |  |  |
| Asian or Asian British | 0.73 (0.59-0.9) | 0.004 | 0.89 (0.68-1.18) | 0.427 | 0.82 (0.56-1.21) | 0.327 |
| Black or Black British | 1.11 (0.83-1.48) | 0.494 | 1.24 (0.86-1.78) | 0.250 | 1.52 (0.97-2.38) | 0.065 |
| Mixed | 0.77 (0.55-1.07) | 0.120 | 0.67 (0.4-1.09) | 0.108 | 0.92 (0.51-1.65) | 0.769 |
| Chinese or other ethnic group | 1.24 (0.92-1.67) | 0.157 | 0.83 (0.55-1.26) | 0.382 | 1.06 (0.63-1.79) | 0.813 |
| Sex (female vs. male) | 2.04 (1.71-2.44) | <0.001 | 2.66 (2.03-3.5) | <0.001 | 1.25 (0.89-1.75) | 0.199 |
| Age, years | 1 (0.99-1.01) | 0.500 | 1.09 (1.07-1.11) | <0.001 | 1.04 (1.02-1.06) | <0.001 |
| Age squared | 1 (1-1) | 0.097 | 1 (1-1) | <0.001 | 1 (1-1) | <0.001 |
| Age X sex | 0.99 (0.99-1) | 0.001 | 0.99 (0.99-1) | 0.021 | 1 (0.99-1.01) | 0.955 |
| 1 / last antidepressant dose, DDD | 0.76 (0.67-0.85) | <0.001 | 1.52 (1.34-1.73) | <0.001 | 0.66 (0.53-0.81) | <0.001 |
| Index year | 0.96 (0.95-0.97) | <0.001 | 0.98 (0.97-1) | 0.031 | 0.94 (0.92-0.96) | <0.001 |
| Last antidepressant dose, DDD | 0.64 (0.58-0.7) | <0.001 | 1.11 (1-1.23) | 0.051 | 1.29 (1.16-1.44) | <0.001 |
| Self-harm (secondary care) | 0.82 (0.68-0.98) | 0.034 | 0.64 (0.48-0.85) | 0.002 | 0.74 (0.53-1.03) | 0.078 |
| First SSRI (vs. citalopram) ^b^ |  |  |  |  |  |  |
| escitalopram | 1.66 (1.36-2.02) | <0.001 | 0.91 (0.69-1.2) | 0.496 | 1.15 (0.83-1.58) | 0.406 |
| fluoxetine | 1.43 (1.32-1.55) | <0.001 | 1.27 (1.14-1.41) | <0.001 | 1.01 (0.88-1.16) | 0.835 |
| paroxetine | 2.21 (1.52-3.21) | <0.001 | 1.44 (0.87-2.36) | 0.154 | 1.38 (0.76-2.5) | 0.293 |
| sertraline | 0.97 (0.88-1.08) | 0.604 | 1.01 (0.89-1.16) | 0.848 | 0.76 (0.63-0.91) | 0.003 |
| Antipsychotics | 0.73 (0.62-0.85) | <0.001 | 0.7 (0.57-0.87) | 0.001 | 1.57 (1.25-1.98) | <0.001 |
| Anxiolytics | 0.69 (0.62-0.77) | <0.001 | 0.68 (0.59-0.79) | <0.001 | 1.03 (0.86-1.23) | 0.735 |
| Glucocorticoids | 0.86 (0.71-1.03) | 0.095 | 1 (0.81-1.24) | 0.987 | 0.76 (0.54-1.08) | 0.133 |
| Hypnotics | 0.56 (0.52-0.61) | <0.001 | 0.51 (0.45-0.57) | <0.001 | 0.74 (0.64-0.86) | <0.001 |
| Opioids | 0.88 (0.79-0.99) | 0.029 | 2.54 (2.24-2.88) | <0.001 | 0.81 (0.66-1) | 0.049 |
| Statins | 1.09 (0.95-1.24) | 0.222 | 1.12 (0.96-1.32) | 0.147 | 0.86 (0.67-1.12) | 0.270 |
| Other analgesics | 1.02 (0.9-1.15) | 0.789 | 1.32 (1.15-1.52) | <0.001 | 0.86 (0.68-1.1) | 0.231 |
| Severe depression | 0.83 (0.74-0.93) | 0.002 | 0.77 (0.66-0.9) | 0.001 | 1.09 (0.9-1.31) | 0.392 |
| Recent cancer | 0.96 (0.69-1.33) | 0.806 | 1.51 (1.04-2.2) | 0.031 | 1.24 (0.69-2.22) | 0.478 |
| Unexplained weight loss | 0.91 (0.77-1.08) | 0.271 | 0.87 (0.69-1.08) | 0.212 | 1.25 (0.93-1.68) | 0.142 |
| Venous thromboembolism | 1.03 (0.73-1.45) | 0.875 | 0.73 (0.47-1.12) | 0.145 | 1.07 (0.59-1.94) | 0.834 |
| Substance misuse disorder | 0.73 (0.6-0.88) | 0.001 | 0.8 (0.6-1.06) | 0.117 | 0.67 (0.46-0.98) | 0.041 |
| Self-harm (primary care) | 0.82 (0.7-0.96) | 0.012 | 0.96 (0.77-1.21) | 0.744 | 1 (0.77-1.32) | 0.974 |
| Rheumatological disease | 0.9 (0.67-1.22) | 0.500 | 0.97 (0.69-1.35) | 0.836 | 1.06 (0.6-1.87) | 0.840 |
| Renal failure | 1.04 (0.86-1.26) | 0.657 | 0.92 (0.73-1.15) | 0.454 | 1.27 (0.87-1.85) | 0.218 |
| Peripheral vascular disease | 1.22 (0.87-1.72) | 0.243 | 1.11 (0.74-1.65) | 0.624 | 1.29 (0.63-2.63) | 0.491 |
| Peptic ulcer disease | 1.04 (0.73-1.49) | 0.810 | 0.99 (0.64-1.53) | 0.955 | 0.83 (0.4-1.72) | 0.615 |
| Parkinson's disease | 0.69 (0.41-1.18) | 0.176 | 0.59 (0.29-1.2) | 0.147 | 0.7 (0.21-2.37) | 0.569 |
| Pancreatitis | 2.17 (1.13-4.17) | 0.021 | 2.33 (1.13-4.81) | 0.022 | 1.81 (0.62-5.24) | 0.276 |
| Palliative / end-of-life care | 0.88 (0.51-1.51) | 0.637 | 1.35 (0.77-2.37) | 0.293 | 0.9 (0.28-2.87) | 0.864 |
| Neuropathic pain | 0.99 (0.88-1.12) | 0.881 | 1.87 (1.63-2.14) | <0.001 | 0.93 (0.74-1.17) | 0.545 |
| Limited mobility | 0.8 (0.71-0.89) | <0.001 | 0.88 (0.76-1.01) | 0.071 | 0.85 (0.69-1.05) | 0.128 |
| Migraine | 0.84 (0.74-0.95) | 0.006 | 1.26 (1.08-1.47) | 0.003 | 0.77 (0.61-0.96) | 0.020 |
| Myocardial infarction | 1.09 (0.81-1.47) | 0.587 | 0.72 (0.49-1.05) | 0.091 | 0.56 (0.25-1.26) | 0.160 |
| Metastatic tumour | 0.53 (0.22-1.3) | 0.167 | 0.47 (0.17-1.3) | 0.146 | 1.17 (0.29-4.78) | 0.827 |
| Leg ulcer | 1.03 (0.69-1.53) | 0.879 | 0.95 (0.6-1.5) | 0.816 | 1.46 (0.73-2.92) | 0.288 |
| Insomnia | 0.75 (0.68-0.83) | <0.001 | 1.21 (1.07-1.37) | 0.003 | 0.82 (0.69-0.98) | 0.031 |
| Indigestion | 1.02 (0.95-1.09) | 0.629 | 1.09 (0.99-1.2) | 0.066 | 1.03 (0.91-1.17) | 0.623 |
| Hypertension | 0.97 (0.87-1.09) | 0.664 | 0.93 (0.81-1.07) | 0.322 | 0.76 (0.61-0.95) | 0.014 |
| Unexpected hospital admission | 0.88 (0.77-1) | 0.050 | 0.99 (0.84-1.18) | 0.931 | 0.95 (0.75-1.22) | 0.699 |
| Hemiplegia | 1.77 (0.56-5.62) | 0.332 | 1.75 (0.45-6.78) | 0.415 |  |  |
| Epilepsy | 0.83 (0.63-1.09) | 0.185 | 0.79 (0.54-1.16) | 0.227 | 0.7 (0.41-1.2) | 0.194 |
| Dyspnoea | 1 (0.89-1.11) | 0.951 | 1.2 (1.05-1.38) | 0.008 | 1.11 (0.91-1.35) | 0.304 |
| Diabetes with complications | 0.78 (0.56-1.08) | 0.129 | 1.28 (0.89-1.83) | 0.189 | 0.72 (0.37-1.4) | 0.338 |
| Diabetes | 0.99 (0.83-1.18) | 0.871 | 0.94 (0.76-1.16) | 0.538 | 1.16 (0.84-1.6) | 0.367 |
| Record of depression scale | 1.05 (0.98-1.13) | 0.174 | 1.05 (0.96-1.16) | 0.270 | 1.01 (0.89-1.14) | 0.867 |
| Dementia | 0.44 (0.32-0.6) | <0.001 | 0.35 (0.23-0.55) | <0.001 | 0.44 (0.19-1.04) | 0.061 |
| Chronic obstructive pulmonary disorder | 1.15 (0.91-1.45) | 0.253 | 0.93 (0.7-1.23) | 0.612 | 1.05 (0.64-1.71) | 0.858 |
| Congestive heart failure | 1.17 (0.82-1.66) | 0.401 | 1.29 (0.85-1.95) | 0.236 | 1.1 (0.47-2.55) | 0.831 |
| Cerebrovascular disease | 1.26 (0.97-1.62) | 0.078 | 1.09 (0.8-1.5) | 0.579 | 1.15 (0.67-1.99) | 0.618 |
| Living in a care home | 1.52 (0.8-2.89) | 0.202 | 0.87 (0.35-2.17) | 0.771 |  |  |
| Cancer | 1.03 (0.87-1.21) | 0.753 | 0.89 (0.73-1.1) | 0.292 | 1.08 (0.79-1.47) | 0.636 |
| Asthma | 0.97 (0.87-1.07) | 0.535 | 1.09 (0.95-1.24) | 0.209 | 1 (0.84-1.19) | 0.963 |
| Anxiety | 1.06 (0.99-1.14) | 0.110 | 1 (0.91-1.11) | 0.951 | 1.08 (0.95-1.23) | 0.226 |
| Appetite loss | 0.84 (0.65-1.1) | 0.203 | 0.73 (0.51-1.04) | 0.082 | 1.01 (0.63-1.61) | 0.963 |
| Angina | 0.92 (0.72-1.19) | 0.547 | 1.06 (0.78-1.42) | 0.719 | 0.87 (0.49-1.57) | 0.654 |
| Anaemia | 0.88 (0.77-1.01) | 0.070 | 0.91 (0.77-1.07) | 0.258 | 1 (0.79-1.28) | 0.973 |
| Alcohol misuse | 0.93 (0.77-1.11) | 0.418 | 0.81 (0.62-1.06) | 0.123 | 0.62 (0.44-0.89) | 0.008 |
| Atrial fibrillation | 0.89 (0.7-1.14) | 0.370 | 0.73 (0.53-0.99) | 0.046 | 0.66 (0.36-1.21) | 0.181 |

DDD defined daily dose, CI confidence interval, SSRI selective serotonin reuptake inhibitor.

^a^ Variables contain imputed data.

^b^ Small numbers (<5) prescribed fluvoxamine were combined with those prescribed citalopram.
