# Supplementary material for: The risk of all-cause and cause-specific mortality in people prescribed mirtazapine: an active comparator cohort study using electronic health records
Source: BMC Med. 2022 Feb 2;20:43. doi: 10.1186/s12916-022-02247-x (PMC8809032; doi:10.1186/s12916-022-02247-x)
Supplement: Supplementary file 3 — Additional file 3: Table S2. Proportion of people with complete data and proportion of deaths recorded in people with complete data. Table S3. Missingness for each variable by study group. [file 12916_2022_2247_MOESM3_ESM.docx]

Additional file 3

**Table S2. Proportion of people with complete data and proportion of deaths recorded in people with complete data.**

|  |  | Number (%) deaths in people with complete data | | | |
| --- | --- | --- | --- | --- | --- |
| group | Number (%) people with complete data | All deaths | Circulatory system deaths | Respiratory system deaths | Cancer deaths |
| Mirtazapine | 1408/5081 (28%) | 74/213 (35%) | 25/51 (49%) | 9/37 (24%) | 19/55 (35%) |
| SSRI | 3777/15032 (25%) | 84/251 (33%) | 30/73 (41%) | 16/45 (36%) | 13/55 (24%) |
| Amitriptyline | 1252/3905 (32%) | 42/107 (39%) | 10/28 (36%) | 6/17 (35%) | 13/38 (34%) |
| Venlafaxine | 357/1580 (23%) | 11/28 (39%) | <5 | <5 | <5 |

SSRI selective serotonin reuptake inhibitor

**Table S3. Missingness for each variable by study group.**

| group | missing alcohol intake | missing BMI | missing ethnicity | missing smoking status | missing Townsend deprivation score | Missing 1+ variable |
| --- | --- | --- | --- | --- | --- | --- |
| Mirtazapine | 59.7% | 30.1% | 24.3% | 3.1% | <0.5% | 72.3% |
| SSRI | 62.2% | 30.2% | 28.2% | 3.2% | <0.5% | 74.9% |
| Amitriptyline | 56.5% | 21.0% | 21.6% | 1.5% | <0.5% | 67.9% |
| Venlafaxine | 65.7% | 28.5% | 29.6% | 3.5% | <0.5% | 77.4% |

SSRI selective serotonin reuptake inhibitor
