# Supplementary material for: The risk of all-cause and cause-specific mortality in people prescribed mirtazapine: an active comparator cohort study using electronic health records
Source: BMC Med. 2022 Feb 2;20:43. doi: 10.1186/s12916-022-02247-x (PMC8809032; doi:10.1186/s12916-022-02247-x)
Supplement: Supplementary file 4 — Additional file 4: Table S4. Full baseline characteristics. [file 12916_2022_2247_MOESM4_ESM.docx]

Additional file 4

**Table S4. Full baseline characteristics.**

|  | **All** | **Mirtazapine** | **SSRI** | **Amitriptyline** | **Venlafaxine** | **Statistic** |
| --- | --- | --- | --- | --- | --- | --- |
| Count | 25,598 | 5,081 | 15,032 | 3,905 | 1,580 |  |
| Age, median (IQR), years | 41 (29-54) | 43 (30-58) | 38 (27-50) | 48 (37-60) | 40 (30-50) | KW chi2(3)=913.1, p=<0.001 |
| Sex, No. (%) |  |  |  |  |  |  |
| Male | 10,617 (41.5%) | 2,609 (51.3%) | 5,937 (39.5%) | 1,346 (34.5%) | 725 (45.9%) |  |
| Female | 14,981 (58.5%) | 2,472 (48.7%) | 9,095 (60.5%) | 2,559 (65.5%) | 855 (54.1%) | chi2(3)=319.9, p=<0.001 |
| Ethnicity, No. (%) ^a^ |  |  |  |  |  |  |
| Asian or Asian British | 457 (2.4%) | 110 (2.9%) | 241 (2.2%) | 83 (2.7%) | 23 (2.1%) |  |
| Black or Black British | 274 (1.5%) | 41 (1.1%) | 159 (1.5%) | 57 (1.9%) | 17 (1.5%) |  |
| Mixed | 167 (0.9%) | 38 (1.0%) | 99 (0.9%) | 19 (0.6%) | 11 (1.0%) |  |
| Chinese or other ethnic group | 219 (1.2%) | 35 (0.9%) | 140 (1.3%) | 32 (1.0%) | 12 (1.1%) |  |
| White | 17,692 (94.1%) | 3,622 (94.2%) | 10,151 (94.1%) | 2,870 (93.8%) | 1,049 (94.3%) | chi2(12)=21.2, p=0.048 |
| Missing ethnicity, No. (%) | 6,789 (26.5%) | 1,235 (24.3%) | 4,242 (28.2%) | 844 (21.6%) | 468 (29.6%) | chi2(3)=91.1, p=<0.001 |
| Deprivation score, No. (%) ^a^ |  |  |  |  |  |  |
| 1 (least deprived) | 4,977 (19.5%) | 893 (17.6%) | 2,944 (19.6%) | 806 (20.6%) | 334 (21.2%) |  |
| 2 | 5,126 (20.0%) | 982 (19.3%) | 2,996 (20.0%) | 778 (19.9%) | 370 (23.4%) |  |
| 3 | 5,514 (21.6%) | 1,066 (21.0%) | 3,241 (21.6%) | 861 (22.0%) | 346 (21.9%) |  |
| 4 | 5,587 (21.8%) | 1,129 (22.2%) | 3,343 (22.3%) | 839 (21.5%) | 276 (17.5%) |  |
| 5 (most deprived) | 4,372 (17.1%) | 1,006 (19.8%) | 2,492 (16.6%) | 621 (15.9%) | 253 (16.0%) | chi2(12)=70.1, p=<0.001 |
| Missing deprivation score, No. (%) | 30 (0.1%) ^c^ | 5 (0.1%) | 16 (0.1%) | <5 | <5 | chi2(3)=4.3, p=0.233 |
| Region, No. (%) |  |  |  |  |  |  |
| North East | 530 (2.1%) | 120 (2.4%) | 289 (1.9%) | 93 (2.4%) | 28 (1.8%) |  |
| North West | 4,524 (17.7%) | 1,277 (25.1%) | 2,419 (16.1%) | 592 (15.2%) | 236 (14.9%) |  |
| Yorkshire & The Humber | 780 (3.0%) | 127 (2.5%) | 493 (3.3%) | 131 (3.4%) | 29 (1.8%) |  |
| East Midlands | 656 (2.6%) | 94 (1.9%) | 439 (2.9%) | 105 (2.7%) | 18 (1.1%) |  |
| West Midlands | 3,188 (12.5%) | 525 (10.3%) | 1,963 (13.1%) | 443 (11.3%) | 257 (16.3%) |  |
| East of England | 2,286 (8.9%) | 359 (7.1%) | 1,327 (8.8%) | 384 (9.8%) | 216 (13.7%) |  |
| South West | 3,572 (14.0%) | 782 (15.4%) | 1,939 (12.9%) | 608 (15.6%) | 243 (15.4%) |  |
| South Central | 3,288 (12.8%) | 562 (11.1%) | 1,957 (13.0%) | 624 (16.0%) | 145 (9.2%) |  |
| London | 2,628 (10.3%) | 479 (9.4%) | 1,622 (10.8%) | 381 (9.8%) | 146 (9.2%) |  |
| South East Coast | 4,146 (16.2%) | 756 (14.9%) | 2,584 (17.2%) | 544 (13.9%) | 262 (16.6%) | chi2(27)=488.9, p=<0.001 |
| BMI, median (IQR) ^a^ | 26.2 (22.8-30.7) | 25.6 (22.4-29.7) | 26.1 (22.7-30.5) | 27.2 (23.5-32.1) | 26.6 (23.1-31.1) | KW chi2(3)=137.3, p=<0.001 |
| Missing BMI, No. (%) | 7,338 (28.7%) | 1,529 (30.1%) | 4,541 (30.2%) | 818 (20.9%) | 450 (28.5%) | chi2(3)=136.3, p=<0.001 |
| Smoking status, No. (%) ^a^ |  |  |  |  |  |  |
| Never | 9,941 (40.0%) | 1,854 (37.6%) | 5,903 (40.6%) | 1,525 (39.7%) | 659 (43.2%) |  |
| Former | 6,762 (27.2%) | 1,300 (26.4%) | 3,853 (26.5%) | 1,197 (31.1%) | 412 (27.0%) |  |
| Current | 8,146 (32.8%) | 1,771 (36.0%) | 4,797 (33.0%) | 1,124 (29.2%) | 454 (29.8%) | chi2(6)=72.2, p=<0.001 |
| Missing smoking status, No. (%) | 749 (2.9%) | 156 (3.1%) | 479 (3.2%) | 59 (1.5%) | 55 (3.5%) | chi2(3)=33.2, p=<0.001 |
| Alcohol intake, No. (%) ^a^ |  |  |  |  |  |  |
| Non-drinker | 3,320 (33.3%) | 647 (31.6%) | 1,945 (34.2%) | 543 (32.0%) | 185 (34.1%) |  |
| Former drinker | 1,438 (14.4%) | 334 (16.3%) | 764 (13.4%) | 272 (16.0%) | 68 (12.5%) |  |
| Occasional drinker | 4,226 (42.4%) | 825 (40.3%) | 2,416 (42.5%) | 753 (44.3%) | 232 (42.8%) |  |
| Moderate drinker | 467 (4.7%) | 104 (5.1%) | 264 (4.6%) | 72 (4.2%) | 27 (5.0%) |  |
| Heavy drinker | 520 (5.2%) | 138 (6.7%) | 293 (5.2%) | 59 (3.5%) | 30 (5.5%) | chi2(12)=41.8, p=<0.001 |
| Missing alcohol intake, No. (%) | 15,627 (61.0%) | 3,033 (59.7%) | 9,350 (62.2%) | 2,206 (56.5%) | 1,038 (65.7%) | chi2(3)=60.8, p=<0.001 |
| Mental health indicators, No. (%) |  |  |  |  |  |  |
| Severe depression ^b^ | 2,421 (9.5%) | 513 (10.1%) | 1,379 (9.2%) | 338 (8.7%) | 191 (12.1%) | chi2(3)=19.5, p=<0.001 |
| Recorded depression scale | 15,727 (61.4%) | 3,035 (59.7%) | 9,256 (61.6%) | 2,444 (62.6%) | 992 (62.8%) | chi2(3)=9.7, p=0.021 |
| Alcohol misuse | 886 (3.5%) | 261 (5.1%) | 461 (3.1%) | 115 (2.9%) | 49 (3.1%) | chi2(3)=53.4, p=<0.001 |
| Anxiety | 7,672 (30.0%) | 1,546 (30.4%) | 4,496 (29.9%) | 1,127 (28.9%) | 503 (31.8%) | chi2(3)=5.4, p=0.142 |
| Eating disorder | 119 (0.5%) | 20 (0.4%) | 78 (0.5%) | 12 (0.3%) | 9 (0.6%) | chi2(3)=4.0, p=0.264 |
| Insomnia | 3,132 (12.2%) | 811 (16.0%) | 1,484 (9.9%) | 647 (16.6%) | 190 (12.0%) | chi2(3)=212.2, p=<0.001 |
| Intellectual disability | 88 (0.3%) | 13 (0.3%) | 59 (0.4%) | 12 (0.3%) | 4 (0.3%) | chi2(3)=2.7, p=0.437 |
| Personality disorder | 140 (0.5%) | 39 (0.8%) | 68 (0.5%) | 22 (0.6%) | 11 (0.7%) | chi2(3)=7.7, p=0.053 |
| Self-harm (primary care) | 1,667 (6.5%) | 419 (8.2%) | 941 (6.3%) | 191 (4.9%) | 116 (7.3%) | chi2(3)=45.3, p=<0.001 |
| Self-harm (secondary care) | 1,082 (4.2%) | 304 (6.0%) | 604 (4.0%) | 104 (2.7%) | 70 (4.4%) | chi2(3)=64.1, p=<0.001 |
| Substance misuse disorder | 655 (2.6%) | 209 (4.1%) | 334 (2.2%) | 76 (1.9%) | 36 (2.3%) | chi2(3)=62.5, p=<0.001 |
| Visited mental health services | 6,475 (25.3%) | 1,653 (32.5%) | 3,576 (23.8%) | 740 (19.0%) | 506 (32.0%) | chi2(3)=280.0, p=<0.001 |
| Medicines, No. (%) |  |  |  |  |  |  |
| Opioids | 4,331 (16.9%) | 833 (16.4%) | 1,845 (12.3%) | 1,473 (37.7%) | 180 (11.4%) | chi2(3)=1,468.2, p=<0.001 |
| Glucocorticoids | 999 (3.9%) | 226 (4.4%) | 476 (3.2%) | 252 (6.5%) | 45 (2.8%) | chi2(3)=98.2, p=<0.001 |
| NSAIDs | 3,749 (14.6%) | 616 (12.1%) | 1,813 (12.1%) | 1,149 (29.4%) | 171 (10.8%) | chi2(3)=806.9, p=<0.001 |
| Other analgesics | 3,328 (13.0%) | 690 (13.6%) | 1,486 (9.9%) | 1,026 (26.3%) | 126 (8.0%) | chi2(3)=774.0, p=<0.001 |
| Statins | 2,961 (11.6%) | 693 (13.6%) | 1,447 (9.6%) | 696 (17.8%) | 125 (7.9%) | chi2(3)=246.7, p=<0.001 |
| Anxiolytics | 2,494 (9.7%) | 677 (13.3%) | 1,223 (8.1%) | 393 (10.1%) | 201 (12.7%) | chi2(3)=134.6, p=<0.001 |
| Antipsychotics | 1,081 (4.2%) | 304 (6.0%) | 496 (3.3%) | 159 (4.1%) | 122 (7.7%) | chi2(3)=118.6, p=<0.001 |
| Hypnotics | 4,141 (16.2%) | 1,262 (24.8%) | 2,006 (13.3%) | 574 (14.7%) | 299 (18.9%) | chi2(3)=385.1, p=<0.001 |
| Comorbidities, No. (%) |  |  |  |  |  |  |
| Abdominal pain | 9,597 (37.5%) | 1,785 (35.1%) | 5,552 (36.9%) | 1,717 (44.0%) | 543 (34.4%) | chi2(3)=90.6, p=<0.001 |
| Inflammatory bowel disease | 169 (0.7%) | 33 (0.6%) | 90 (0.6%) | 40 (1.0%) | 6 (0.4%) | chi2(3)=10.7, p=0.014 |
| Indigestion | 8,520 (33.3%) | 1,704 (33.5%) | 4,787 (31.8%) | 1,533 (39.3%) | 496 (31.4%) | chi2(3)=79.4, p=<0.001 |
| Liver disease (mild) | 55 (0.2%) ^c^ | 15 (0.3%) | 22 (0.1%) | 10 (0.3%) | <5 | chi2(3)=5.3, p=0.149 |
| Liver disease (moderate) | 15 (0.0%) ^c^ | <5 | 8 (0.1%) | <5 | <5 | chi2(3)=0.7, p=0.883 |
| Obesity | 1,557 (6.1%) | 262 (5.2%) | 847 (5.6%) | 345 (8.8%) | 103 (6.5%) | chi2(3)=65.2, p=<0.001 |
| Pancreatitis | 92 (0.4%) | 12 (0.2%) | 51 (0.3%) | 24 (0.6%) | 5 (0.3%) | chi2(3)=9.5, p=0.023 |
| Peptic ulcer disease | 205 (0.8%) | 53 (1.0%) | 101 (0.7%) | 42 (1.1%) | 9 (0.6%) | chi2(3)=11.7, p=0.009 |
| Renal failure | 896 (3.5%) | 242 (4.8%) | 410 (2.7%) | 202 (5.2%) | 42 (2.7%) | chi2(3)=86.2, p=<0.001 |
| Anaemia | 1,698 (6.6%) | 362 (7.1%) | 892 (5.9%) | 348 (8.9%) | 96 (6.1%) | chi2(3)=47.4, p=<0.001 |
| Atrial fibrillation | 432 (1.7%) | 136 (2.7%) | 200 (1.3%) | 83 (2.1%) | 13 (0.8%) | chi2(3)=53.1, p=<0.001 |
| Angina | 481 (1.9%) | 127 (2.5%) | 216 (1.4%) | 123 (3.1%) | 15 (0.9%) | chi2(3)=68.2, p=<0.001 |
| Cerebrovascular disease | 444 (1.7%) | 111 (2.2%) | 224 (1.5%) | 92 (2.4%) | 17 (1.1%) | chi2(3)=24.2, p=<0.001 |
| Congestive heart failure | 226 (0.9%) | 59 (1.2%) | 104 (0.7%) | 56 (1.4%) | 7 (0.4%) | chi2(3)=27.8, p=<0.001 |
| Diabetes | 1,418 (5.5%) | 314 (6.2%) | 677 (4.5%) | 352 (9.0%) | 75 (4.7%) | chi2(3)=126.8, p=<0.001 |
| Diabetes with complications | 332 (1.3%) | 77 (1.5%) | 137 (0.9%) | 105 (2.7%) | 13 (0.8%) | chi2(3)=81.2, p=<0.001 |
| Hypertension | 3,071 (12.0%) | 726 (14.3%) | 1,516 (10.1%) | 694 (17.8%) | 135 (8.5%) | chi2(3)=218.5, p=<0.001 |
| Myocardial infarction | 309 (1.2%) | 82 (1.6%) | 162 (1.1%) | 58 (1.5%) | 7 (0.4%) | chi2(3)=19.4, p=<0.001 |
| Peripheral vascular disease | 246 (1.0%) | 59 (1.2%) | 118 (0.8%) | 59 (1.5%) | 10 (0.6%) | chi2(3)=21.2, p=<0.001 |
| Venous thromboembolism | 234 (0.9%) | 52 (1.0%) | 122 (0.8%) | 45 (1.2%) | 15 (0.9%) | chi2(3)=4.9, p=0.181 |
| Appetite loss | 371 (1.4%) | 99 (1.9%) | 192 (1.3%) | 56 (1.4%) | 24 (1.5%) | chi2(3)=12.0, p=0.007 |
| Living in a care home | 60 (0.2%) ^c^ | 21 (0.4%) | 24 (0.2%) | 8 (0.2%) | <5 | chi2(3)=15.4, p=0.002 |
| Hemiplegia | 25 (0.1%) ^c^ | <5 | 14 (0.1%) | 6 (0.2%) | <5 | chi2(3)=3.1, p=0.376 |
| Leg ulcer | 178 (0.7%) | 42 (0.8%) | 80 (0.5%) | 45 (1.2%) | 11 (0.7%) | chi2(3)=18.9, p=<0.001 |
| Palliative/end-of-life care | 100 (0.4%) ^c^ | 30 (0.6%) | 32 (0.2%) | 33 (0.8%) | <5 | chi2(3)=39.3, p=<0.001 |
| Reduced mobility | 2,803 (11.0%) | 731 (14.4%) | 1,370 (9.1%) | 560 (14.3%) | 142 (9.0%) | chi2(3)=165.8, p=<0.001 |
| Unexplained weight loss | 942 (3.7%) | 238 (4.7%) | 489 (3.3%) | 151 (3.9%) | 64 (4.1%) | chi2(3)=23.2, p=<0.001 |
| Unexpected hospital admission | 1,602 (6.3%) | 401 (7.9%) | 808 (5.4%) | 300 (7.7%) | 93 (5.9%) | chi2(3)=57.0, p=<0.001 |
| Asthma | 3,522 (13.8%) | 676 (13.3%) | 2,012 (13.4%) | 620 (15.9%) | 214 (13.5%) | chi2(3)=17.5, p=0.001 |
| Chronic obstructive pulmonary disease | 584 (2.3%) | 145 (2.9%) | 276 (1.8%) | 140 (3.6%) | 23 (1.5%) | chi2(3)=55.4, p=<0.001 |
| Dyspnoea | 3,325 (13.0%) | 709 (14.0%) | 1,681 (11.2%) | 756 (19.4%) | 179 (11.3%) | chi2(3)=191.7, p=<0.001 |
| Sleep apnoea | 201 (0.8%) | 36 (0.7%) | 97 (0.6%) | 53 (1.4%) | 15 (0.9%) | chi2(3)=21.1, p=<0.001 |
| Acquired immune deficiency syndrome/AIDS | <5 | <5 | <5 | <5 | <5 | chi2(3)=1.4, p=0.704 |
| Cancer | 1,392 (5.4%) | 334 (6.6%) | 683 (4.5%) | 299 (7.7%) | 76 (4.8%) | chi2(3)=74.7, p=<0.001 |
| Recent cancer | 332 (1.3%) | 79 (1.6%) | 140 (0.9%) | 93 (2.4%) | 20 (1.3%) | chi2(3)=54.2, p=<0.001 |
| Metastatic tumour | 35 (0.1%) ^c^ | 12 (0.2%) | 10 (0.1%) | 8 (0.2%) | <5 | chi2(3)=11.3, p=0.010 |
| Dementia | 224 (0.9%) | 114 (2.2%) | 74 (0.5%) | 30 (0.8%) | 6 (0.4%) | chi2(3)=140.1, p=<0.001 |
| Epilepsy | 318 (1.2%) | 82 (1.6%) | 172 (1.1%) | 47 (1.2%) | 17 (1.1%) | chi2(3)=7.3, p=0.063 |
| Fibromyalgia | 575 (2.2%) | 85 (1.7%) | 260 (1.7%) | 204 (5.2%) | 26 (1.6%) | chi2(3)=186.2, p=<0.001 |
| Huntington’s disease | 10 (0.0%) ^c^ | <5 | <5 | <5 | <5 | chi2(3)=1.5, p=0.683 |
| Migraine | 2,128 (8.3%) | 393 (7.7%) | 1,182 (7.9%) | 447 (11.4%) | 106 (6.7%) | chi2(3)=61.9, p=<0.001 |
| Multiple sclerosis | 60 (0.2%) ^c^ | 10 (0.2%) | 16 (0.1%) | 27 (0.7%) | <5 | chi2(3)=48.0, p=<0.001 |
| Neuropathic pain | 2,376 (9.3%) | 441 (8.7%) | 1,064 (7.1%) | 762 (19.5%) | 109 (6.9%) | chi2(3)=585.0, p=<0.001 |
| Parkinson’s disease | 80 (0.3%) ^c^ | 29 (0.6%) | 31 (0.2%) | 13 (0.3%) | <5 | chi2(3)=17.8, p=<0.001 |
| Rheumatological | 325 (1.3%) | 76 (1.5%) | 144 (1.0%) | 89 (2.3%) | 16 (1.0%) | chi2(3)=46.3, p=<0.001 |
| First SSRI ^d^ |  |  |  |  |  |  |
| Citalopram | 13,184 (51.5%) | 2,858 (56.2%) | 7,352 (48.9%) | 2,112 (54.1%) | 862 (54.6%) |  |
| Escitalopram | 902 (3.5%) | 139 (2.7%) | 597 (4.0%) | 104 (2.7%) | 62 (3.9%) |  |
| Fluoxetine | 7,813 (30.5%) | 1,236 (24.3%) | 5,047 (33.6%) | 1,089 (27.9%) | 441 (27.9%) |  |
| Paroxetine | 296 (1.2%) | 34 (0.7%) | 212 (1.4%) | 33 (0.8%) | 17 (1.1%) |  |
| Sertraline | 3,403 (13.3%) | 814 (16.0%) | 1,824 (12.1%) | 567 (14.5%) | 198 (12.5%) | chi2(12)=269.3, p=<0.001 |
| Index year, median (IQR) | 2011 (2008-2013) | 2011 (2009-2014) | 2011 (2008-2013) | 2011 (2009-2013) | 2010 (2008-2013) | KW chi2(3)=124.7, p=<0.001 |
| Most recent antidepressant dose at index, median (IQR), DDD | 1 (1-1) | 1 (1-1.5) | 1 (1-1) | 1 (1-1) | 1 (1-2) | KW chi2(3)=269.6, p=<0.001 |
| Current antidepressant dose at index, median (IQR), DDD | 1 (0-1) | 1 (0-1) | 1 (0-1) | 1 (0-1) | 1 (0-1.9) | KW chi2(3)=312.8, p=<0.001 |
| Time between starting first and second antidepressant, median (IQR), weeks | 19 (6.3-76.3) | 16.9 (6-66.7) | 15.6 (5.3-62.6) | 46.9 (12.3-137.4) | 27.1 (10.4-87) | KW chi2(3)=804.5, p=<0.001 |
| First antidepressant still active at index, No. (%) | 16,459 (64.3%) | 3,492 (68.7%) | 8,982 (59.8%) | 2,918 (74.7%) | 1,067 (67.5%) | chi2(3)=370.8, p=<0.001 |

Differences between the antidepressant groups were compared using chi-squared or Kruskal-Wallis tests. SSRI selective serotonin reuptake inhibitor, IQR interquartile range, KW Kruskal-Wallis test, chi2() Chi-squared test (degrees of freedom), BMI body mass index.

^a^ Counts and percentages do not include missing values.

^b^ Severe depression: record of severe depression or depression with psychosis, scoring 15 or above on the Patient Health Questionnaire-9 (PHQ-9) scale, or scoring 16 or above on the Hospital Anxiety and Depression (HAD) scale.

^c^ Value rounded to mask small numbers.

^d^ Small numbers (<5) prescribed fluvoxamine were combined with those prescribed citalopram.
