# Supplementary material for: The risk of all-cause and cause-specific mortality in people prescribed mirtazapine: an active comparator cohort study using electronic health records
Source: BMC Med. 2022 Feb 2;20:43. doi: 10.1186/s12916-022-02247-x (PMC8809032; doi:10.1186/s12916-022-02247-x)
Supplement: Supplementary file 5 — Additional file 5. Sensitivity analyses: rates of all-cause mortality and mortality due to neoplasms. Table S5. Rates of all-cause mortality per 1000 person-years according to period of follow-up. Table S6. Sensitivity analysis – Rates of death due to neoplasms per 1000 person-years according to period of follow-up. Table S9. Sensitivity analysis – Rates of all-cause mortality per 1000 person-years after altering study inclusion criteria. [file 12916_2022_2247_MOESM5_ESM.docx]

Additional file 5 – Sensitivity analyses: rates of all-cause mortality and mortality due to neoplasms.

**Table S5. Rates of all-cause mortality per 1000 person-years according to period of follow-up.**

| **Group** | **Crude mortality rate (95% CI)** | **Standardised mortality rate (95% CI)** | **Excess risk (95% CI)** |
| --- | --- | --- | --- |
| Follow-up less than 2 years (429 deaths in 37,209 person-years) |  |  |  |
| All | 11.5 (10.5-12.7) | 11.5 (10.5-12.7) |  |
| Mirtazapine | 24.7 (21.1-28.9) | 15.8 (13.3-18.7) | reference |
| SSRI | 7.1 (6.1-8.2) | 9.1 (7.8-10.7) | -6.7 (-8.3--5.1) |
| Amitriptyline | 18.8 (15.2-23.1) | 14.7 (11.6-18.3) | -1.1 (-2.9-0.6) |
| Venlafaxine | 6.6 (4.2-10.3) | 10.5 (6.1-16.6) | -5.3 (-6.9--3.6) |
| Follow-up greater than or equal to 2 years (170 deaths in 23,652 person-years) |  |  |  |
| All | 7.2 (6.2-8.4) | 7.0 (6.0-8.1) |  |
| Mirtazapine | 14.7 (11.3-19.1) | 9.7 (6.9-13.2) | reference |
| SSRI | 5.7 (4.7-7.1) | 7.2 (5.8-8.9) | -2.5 (-3.8--1.2) |
| Amitriptyline | 6.9 (4.3-10.9) | 4.2 (2.4-6.9) | -5.5 (-6.7--4.3) |
| Venlafaxine | 4.3 (2.2-8.3) | 6.4 (2.8-12.3) | -3.3 (-4.6--2.0) |

CI confidence interval, SSRI selective serotonin reuptake inhibitor. Standardised mortality rates are age-sex standardised using the structure of the overall study population.

**Table S6. Sensitivity analysis – Rates of death due to neoplasms per 1000 person-years according to period of follow-up**

| **Group** | **Crude mortality rate (95% CI)** | **Standardised mortality rate (95% CI)** | **Excess risk (95% CI)** |
| --- | --- | --- | --- |
| **Follow-up less than 2 years (119 deaths in 37,209 person-years)** |  |  |  |
| All | 3.2 (2.7-3.8) | 3.2 (2.6-3.8) |  |
| Mirtazapine | 6.4 (4.7-8.8) | 4.1 (2.9-5.7) | reference |
| SSRI | 1.5 (1.1-2.1) | 1.9 (1.3-2.7) | -2.2 (-3.0--1.4) |
| Amitriptyline | 7.8 (5.7-10.8) | 6.1 (4.2-8.6) | 2.0 (1.0-3.0) |
| Venlafaxine | 2.1 (0.9-4.6) | 2.8 (1.0-6.4) | -1.3 (-2.1--0.4) |
| **Follow-up greater than or equal to 2 years (37 deaths in 23,652 person-years)** |  |  |  |
| All | 1.6 (1.1-2.2) | 1.5 (1.1-2.1) |  |
| Mirtazapine | 3.7 (2.2-6.2) | 3.0 (1.4-5.5) | reference |
| SSRI | 1.3 (0.9-2.0) | 1.6 (0.9-2.4) | -1.4 (-2.1--0.7) |
| Amitriptyline | 0.4 (0.1-2.7) | 0.3 (0.0-1.8) | -2.7 (-3.3--2.1) |
| Venlafaxine | 1.0 (0.2-3.8) | 1.0 (0.1-4.2) | -1.9 (-2.6--1.3) |

CI confidence interval, SSRI selective serotonin reuptake inhibitor. Standardised mortality rates are age-sex standardised using the structure of the overall study population.

**Table S9. Sensitivity analysis – Rates of all-cause mortality per 1000 person-years after altering study inclusion criteria**

| **Group** | **Crude mortality rate (95% CI)** | **Standardised mortality rate (95% CI)** | **Excess risk (95% CI)** |
| --- | --- | --- | --- |
| Include people with baseline records for schizophrenia or bipolar disorder (605 deaths) |  |  |  |
| All | 16.2 (14.9-17.5) | 16.2 (14.9-17.5) |  |
| Mirtazapine | 34.0 (29.8-38.9) | 21.9 (18.9-25.4) | reference |
| SSRI | 10.8 (9.5-12.2) | 13.8 (12.1-15.6) | -8.2 (-10.1--6.3) |
| Amitriptyline | 22.4 (18.6-27.1) | 17.5 (14.2-21.4) | -4.4 (-6.4--2.4) |
| Venlafaxine | 9.5 (6.6-13.8) | 18.8 (11.3-29.0) | -3.1 (-5.2--1.1) |
| Exclude people with baseline records for self-harm (584 deaths) |  |  |  |
| All | 16.8 (15.5-18.2) | 16.8 (15.4-18.2) |  |
| Mirtazapine | 35.8 (31.3-41.0) | 23.0 (19.7-26.7) | reference |
| SSRI | 11.2 (9.9-12.7) | 14.4 (12.6-16.3) | -8.6 (-10.6--6.6) |
| Amitriptyline | 22.8 (18.8-27.7) | 18.0 (14.6-22.1) | -4.9 (-7.1--2.8) |
| Venlafaxine | 10.1 (6.9-14.7) | 19.9 (11.8-30.7) | -3.1 (-5.3--0.9) |
| Exclude people on the palliative care/end-of-life pathway at baseline (547 deaths) |  |  |  |
| All | 14.7 (13.5-16.0) | 14.7 (13.5-16.0) |  |
| Mirtazapine | 30.2 (26.2-34.8) | 19.6 (16.7-22.9) | reference |
| SSRI | 10.4 (9.2-11.8) | 13.2 (11.6-15.0) | -6.4 (-8.3--4.6) |
| Amitriptyline | 19.1 (15.5-23.4) | 14.9 (11.9-18.6) | -4.7 (-6.6--2.8) |
| Venlafaxine | 8.7 (5.9-12.9) | 16.5 (9.6-26.0) | -3.2 (-5.1--1.2) |

CI confidence interval, SSRI selective serotonin reuptake inhibitor. Standardised mortality rates are age-sex standardised using the structure of the overall study population.
