# Supplementary material for: The risk of all-cause and cause-specific mortality in people prescribed mirtazapine: an active comparator cohort study using electronic health records
Source: BMC Med. 2022 Feb 2;20:43. doi: 10.1186/s12916-022-02247-x (PMC8809032; doi:10.1186/s12916-022-02247-x)
Supplement: Supplementary file 6 — Additional file 6: Table S7. Cox regression, risk of all-cause mortality after accounting for current antidepressant dose. [file 12916_2022_2247_MOESM6_ESM.docx]

Additional file 6

Table S7. Cox regression, risk of all-cause mortality after accounting for current antidepressant dose.

|  | **Age-sex adjusted** | **Multivariable adjusted model** | **PS weighted model** |
| --- | --- | --- | --- |
| *Time < 2 years from starting treatment* | | | |
| Mirtazapine / SSRI | 1.54 (1.16-2.06) | 1.46 (1.08-1.97) | 1.39 (1.02-1.90) |
| Mirtazapine / Amitriptyline | 1.22 (0.87-1.69) | 1.31 (0.92-1.87) | 1.07 (0.72-1.59) |
| Mirtazapine / Venlafaxine | 0.81 (0.43-1.51) | 0.66 (0.34-1.26) | 1.11 (0.48-2.57) |
| *Time 2+ years from starting treatment* | | | |
| Mirtazapine / SSRI | 1.23 (0.84-1.80) | 1.14 (0.76-1.70) | 1.31 (0.89-1.92) |
| Mirtazapine / Amitriptyline | 2.27 (1.28-4.05) | 2.14 (1.18-3.88) | 2.40 (1.29-4.45) |
| Mirtazapine / Venlafaxine | 0.88 (0.40-1.94) | 0.74 (0.33-1.66) | 2.34 (0.76-7.16) |
|  |  |  |  |
| Mirtazapine current dose, DDD | 1.07 (0.82-1.41) | 1.02 (0.77-1.35) | *1.41 (1.08-1.84) a* |
| SSRI current dose, DDD | 1.04 (0.92-1.16) | 1.01 (0.89-1.14) | *1.06 (0.94-1.20) a* |
| Amitriptyline current dose, DDD | 2.05 (1.16-3.62) | 2.35 (1.25-4.41) | *1.53 (0.90-2.63) a* |
| venlafaxine current dose, DDD | 0.59 (0.33-1.05) | 0.60 (0.33-1.12) | *1.26 (0.54-2.94) a* |

PS propensity score, SSRI selective serotonin reuptake inhibitor, DDD defined daily dose. Analyses performed using Cox regression. The multivariable adjusted model included the variables used to estimate propensity scores. The PS weighted model applied inverse probability of treatment weights.

^a^ These estimates will not be well adjusted for baseline characteristics: the propensity score balances probability of being prescribed each antidepressant.
