# Supplementary material for: The risk of all-cause and cause-specific mortality in people prescribed mirtazapine: an active comparator cohort study using electronic health records
Source: BMC Med. 2022 Feb 2;20:43. doi: 10.1186/s12916-022-02247-x (PMC8809032; doi:10.1186/s12916-022-02247-x)
Supplement: Supplementary file 7 — Additional file 7: Table S8. Cox regression, risk of all-cause mortality according to switching to or augmenting SSRI with mirtazapine. [file 12916_2022_2247_MOESM7_ESM.docx]

Additional file 7

Table S8. Cox regression, risk of all-cause mortality according to switching to or augmenting SSRI with mirtazapine.

|  | **Unadjusted** | **Age-sex adjusted** | **Multivariable adjusted model** |
| --- | --- | --- | --- |
| Switch/Augment | 0.84 (0.56-1.27) | 1.07 (0.71-1.61) | 1.35 (0.81-2.27) |
| Age at index, years | -- | 1.09 (1.08-1.10) | 1.05 (0.96-1.14) |
| Sex (female/male) | -- | 0.66 (0.48-0.92) | 1.08 (0.18-6.69) |

SSRI selective serotonin reuptake inhibitor. The multivariable adjusted model included the variables used to estimate propensity scores. People were classified as switch (3328 people) or augment (752 people) based on whether they had an active SSRI prescription on the date that was 84 days after their index date. Follow-up start was reset to this later date.
