# Supplementary material for: The risk of all-cause and cause-specific mortality in people prescribed mirtazapine: an active comparator cohort study using electronic health records
Source: BMC Med. 2022 Feb 2;20:43. doi: 10.1186/s12916-022-02247-x (PMC8809032; doi:10.1186/s12916-022-02247-x)
Supplement: Supplementary file 8 — Additional file 8. Sensitivity analyses for all outcomes: varying the survival analysis models. Table S10. Sensitivity analyses for all-cause mortality: varying the survival analysis models. Table S11. Sensitivity analyses for deaths due to diseases of the circulatory system: varying the survival analysis models. Table S12. Sensitivity analyses for deaths due to diseases of the respiratory system: varying the survival analysis models. Table S13. Sensitivity analyses for deaths due to neoplasms: varying the survival analysis models. [file 12916_2022_2247_MOESM8_ESM.docx]

Additional file 8 – Sensitivity analyses for all outcomes: varying the survival analysis models.

**Table S10. Sensitivity analyses for all-cause mortality: varying the survival analysis models.**

|  |  |  |  | **Follow-up <2years ^a^**  **Hazard Ratio (95% confidence interval)** | | | **Follow-up 2+ years**  **Hazard Ratio (95% confidence interval)** | | |
| --- | --- | --- | --- | --- | --- | --- | --- | --- | --- |
| **Analysis, Model** | **People, No.** | **Deaths, No.** | **Follow-up, years** | **Mirtazapine/**  **SSRI** | **Mirtazapine/**  **Amitriptyline** | **Mirtazapine/**  **Venlafaxine** | **Mirtazapine/**  **SSRI** | **Mirtazapine/**  **Amitriptyline** | **Mirtazapine/**  **Venlafaxine** |
| **Main analysis, including results for ‘fully-adjusted’ multivariable regression** | | | | | | |  |  |  |
| Unadjusted | 25,598 | 599 | 37,209 | 3.19 (2.56-3.97) | 1.35 (1.04-1.75) | 3.10 (1.92-4.99) | 2.68 (1.92-3.75) | 2.28 (1.34-3.87) | 3.64 (1.80-7.35) |
| Age-sex adjusted | 25,598 | 599 | 37,209 | 1.58 (1.27-1.97) | 1.12 (0.86-1.45) | 1.26 (0.78-2.03) | 1.26 (0.90-1.76) | 2.01 (1.18-3.41) | 1.33 (0.66-2.70) |
| Fully adjusted | 25,598 | 599 | 37,209 | 1.47 (1.16-1.85) | 1.15 (0.86-1.52) | 0.97 (0.59-1.58) | 1.15 (0.80-1.64) | 1.81 (1.05-3.13) | 1.04 (0.50-2.14) |
| Weighted | 25,598 | 599 | 37,209 | 1.62 (1.28-2.06) | 1.18 (0.85-1.63) | 1.11 (0.60-2.05) | 1.51 (1.04-2.19) | 2.59 (1.38-4.86) | 2.35 (1.02-5.44) |
|  |  |  |  |  |  |  |  |  |  |
| **Complete case analysis** | | | | | | |  |  |  |
| Weighted | 6,794 | 211 | 9,339 | 1.37 (0.91-2.05) | 1.36 (0.79-2.36) | 0.95 (0.35-2.52) | 1.80 (0.96-3.35) | 1.44 (0.66-3.12) | 2.36 (0.62-9.00) |
|  |  |  |  |  |  |  |  |  |  |
| **Include only variables with complete data in models** | | | | | | |  |  |  |
| Weighted | 25,598 | 599 | 37,209 | 1.67 (1.32-2.12) | 1.21 (0.88-1.66) | 1.23 (0.68-2.23) | 1.52 (1.05-2.19) | 2.73 (1.47-5.06) | 2.52 (1.12-5.71) |
|  |  |  |  |  |  |  |  |  |  |
| **Include all baseline covariates** **in models ^b^** | | | |  |  |  |  |  |  |
| Fully adjusted | 25,598 | 599 | 37,209 | 1.52 (1.19-1.92) | 1.19 (0.89-1.58) | 1.01 (0.62-1.66) | 1.16 (0.81-1.66) | 1.82 (1.05-3.17) | 1.12 (0.54-2.34) |
| Weighted | 25,598 | 599 | 37,209 | 1.66 (1.29-2.14) | 1.19 (0.83-1.70) | 1.10 (0.56-2.14) | 1.56 (1.07-2.27) | 2.53 (1.35-4.75) | 2.41 (1.03-5.68) |
|  |  |  |  |  |  |  |  |  |  |
| **Restrict to people aged 16-64 years at baseline** | | | | | | |  |  |  |
| Age-sex adjusted | 22,380 | 153 | 32,490 | 2.66 (1.65-4.30) | 1.15 (0.68-1.94) | 1.22 (0.58-2.55) | 1.91 (1.01-3.61) | 2.00 (0.77-5.17) | 3.29 (0.75-14.42) |
| Fully adjusted | 22,380 | 153 | 32,490 | 2.09 (1.24-3.53) | 1.05 (0.59-1.88) | 1.23 (0.54-2.77) | 1.48 (0.76-2.89) | 2.38 (0.87-6.51) | 3.08 (0.67-14.13) |
|  |  |  |  |  |  |  |  |  |  |
| **Restrict to people aged 65-100 years at baseline** | | | | | | |  |  |  |
| Age-sex adjusted | 3,218 | 446 | 4,719 | 1.36 (1.06-1.74) | 1.16 (0.86-1.58) | 1.44 (0.76-2.75) | 1.06 (0.71-1.58) | 2.12 (1.11-4.04) | 0.90 (0.40-2.02) |
| Fully adjusted | 3,218 | 446 | 4,719 | 1.34 (1.03-1.75) | 1.16 (0.83-1.62) | 1.01 (0.52-1.97) | 1.02 (0.67-1.56) | 1.81 (0.93-3.55) | 0.58 (0.25-1.36) |
|  |  |  |  |  |  |  |  |  |  |
| **No risk carry-over window** | | | |  |  |  |  |  |  |
| Age-sex adjusted | 25,598 | 359 | 22,050 | 1.47 (1.10-1.96) | 0.85 (0.60-1.20) | 1.65 (0.80-3.41) | 1.23 (0.81-1.85) | 2.00 (0.99-4.02) | 1.26 (0.50-3.22) |
| Fully adjusted | 25,598 | 359 | 22,050 | 1.36 (1.00-1.86) | 0.86 (0.58-1.25) | 1.15 (0.54-2.44) | 1.13 (0.73-1.76) | 1.96 (0.96-4.04) | 0.97 (0.37-2.54) |
|  |  |  |  |  |  |  |  |  |  |
| **30-day risk carry-over window** | | | |  |  |  |  |  |  |
| Age-sex adjusted | 25,598 | 467 | 27,827 | 1.56 (1.21-2.01) | 0.96 (0.71-1.31) | 1.24 (0.73-2.13) | 1.23 (0.86-1.77) | 2.15 (1.14-4.04) | 1.63 (0.70-3.82) |
| Fully adjusted | 25,598 | 467 | 27,827 | 1.49 (1.14-1.94) | 1.00 (0.72-1.39) | 0.88 (0.53-1.46) | 1.18 (0.80-1.73) | 1.84 (1.03-3.28) | 1.62 (0.83-3.15) |
|  |  |  |  |  |  |  |  |  |  |
| **Do not censor at end of antidepressant treatment** | | | |  |  |  |  |  |  |
| Age-sex adjusted | 25,598 | 810 | 65,073 | 1.47 (1.20-1.80) | 1.20 (0.94-1.52) | 1.33 (0.84-2.11) | 1.26 (0.96-1.65) | 1.61 (1.15-2.27) | 1.22 (0.68-2.20) |
| Fully adjusted | 25,598 | 810 | 65,073 | 1.33 (1.08-1.65) | 1.17 (0.91-1.51) | 1.01 (0.63-1.62) | 1.12 (0.84-1.48) | 1.55 (1.09-2.20) | 0.96 (0.53-1.75) |
|  |  |  |  |  |  |  |  |  |  |
| **Censor follow-up after 1 year** | | | |  |  |  |  |  |  |
| Age-sex adjusted | 25,598 | 340 | 8,179 | 1.80 (1.40-2.31) | 1.12 (0.84-1.49) | 1.30 (0.76-2.23) |  |  |  |
| Fully adjusted | 25,598 | 340 | 8,179 | 1.64 (1.25-2.15) | 1.12 (0.82-1.53) | 0.99 (0.57-1.73) |  |  |  |
|  |  |  |  |  |  |  |  |  |  |
| **Censor follow-up after 5 years** | | | |  |  |  |  |  |  |
| Age-sex adjusted | 25,598 | 562 | 24,886 | 1.47 (1.22-1.78) | 1.26 (0.99-1.61) | 1.18 (0.79-1.75) |  |  |  |
| Fully adjusted | 25,598 | 562 | 24,886 | 1.36 (1.11-1.68) | 1.25 (0.96-1.63) | 0.88 (0.58-1.34) |  |  |  |
|  |  |  |  |  |  |  |  |  |  |
| **Exclude people with cancer at baseline** | | | |  |  |  |  |  |  |
| Age-sex adjusted | 24,206 | 442 | 35,397 | 1.49 (1.15-1.92) | 1.37 (0.98-1.90) | 1.20 (0.70-2.06) | 1.24 (0.85-1.80) | 2.11 (1.16-3.85) | 1.69 (0.72-3.96) |
| Fully adjusted | 24,206 | 442 | 35,397 | 1.50 (1.13-1.98) | 1.29 (0.91-1.83) | 0.93 (0.56-1.55) | 1.22 (0.82-1.81) | 1.96 (1.11-3.48) | 1.41 (0.73-2.72) |
|  |  |  |  |  |  |  |  |  |  |
| **Exclude people with self-harm at baseline** | | | |  |  |  |  |  |  |
| Age-sex adjusted | 23,653 | 584 | 34,818 | 1.61 (1.29-2.02) | 1.14 (0.87-1.48) | 1.29 (0.79-2.11) | 1.25 (0.89-1.75) | 2.13 (1.24-3.68) | 1.31 (0.65-2.66) |
| Fully adjusted | 23,653 | 584 | 34,818 | 1.51 (1.19-1.92) | 1.18 (0.89-1.58) | 1.00 (0.61-1.66) | 1.14 (0.80-1.63) | 1.95 (1.11-3.41) | 1.03 (0.50-2.14) |

SSRI selective serotonin reuptake inhibitor. ‘Fully adjusted’ models used multivariable regression, ‘weighted’ models used inverse probability of treatment weighting.

^a^ All follow-up in the models that did not include the interaction term.

^b^ Some variables were excluded as the models did not converge. Excluded variables were: AIDs, moderate liver disease, and Huntington’s disease.

**Table S11. Sensitivity analyses for deaths due to diseases of the circulatory system: varying the survival analysis models.**

|  |  |  |  | **Hazard ratios ^a^ (95% confidence interval)** | | |  |
| --- | --- | --- | --- | --- | --- | --- | --- |
| **Analysis, Model** | **People, No.** | **Deaths, No.** | **Follow-up, years** | **Mirtazapine/**  **SSRI** | **Mirtazapine/**  **Amitriptyline** | **Mirtazapine/**  **Venlafaxine** |  |
| **Main analysis, including results for ‘fully-adjusted’ multivariable regression (Fine-Gray regression)** | | | | | | | |
| Unadjusted | 25,598 | 159 | 37,209 | 2.42 (1.70-3.46) | 1.35 (0.85-2.16) | 3.07 (1.39-6.77) |  |
| Age-sex adjusted | 25,598 | 159 | 37,209 | 1.10 (0.76-1.58) | 1.04 (0.64-1.67) | 1.09 (0.49-2.43) |  |
| Fully adjusted | 25,598 | 159 | 37,209 | 1.22 (0.81-1.84) | 1.07 (0.63-1.82) | 0.99 (0.43-2.27) |  |
| Weighted | 25,598 | 159 | 37,209 | 1.41 (0.96-2.08) | 1.11 (0.65-1.88) | 0.74 (0.27-2.01) |  |
|  |  |  |  |  |  |  |  |
| **Complete case analysis** | | | | | | |  |
| Weighted | 6,794 | 68 | 9,339 | 1.63 (0.92-2.86) | 1.52 (0.61-3.80) | -- |  |
|  |  |  |  |  |  |  |  |
| **Include only variables with complete data in models** | | | | | | |  |
| Weighted | 25,598 | 159 | 37,209 | 1.41 (0.96-2.06) | 1.09 (0.64-1.85) | 0.82 (0.31-2.19) |  |
|  |  |  |  |  |  |  |  |
| **Repeat main analysis using Cox regression instead of Fine-Gray regression** | | | | | | | |
| Unadjusted | 25,598 | 159 | 37,209 | 2.50 (1.75-3.57) | 1.38 (0.87-2.18) | 3.18 (1.44-7.01) |  |
| Age-sex adjusted | 25,598 | 159 | 37,209 | 1.14 (0.80-1.64) | 1.11 (0.70-1.76) | 1.12 (0.51-2.48) |  |
| Fully adjusted | 25,598 | 159 | 37,209 | 1.31 (0.88-1.94) | 1.14 (0.68-1.90) | 1.01 (0.44-2.33) |  |
| Weighted | 25,598 | 159 | 37,209 | 1.43 (0.97-2.10) | 1.12 (0.66-1.89) | 0.75 (0.28-2.05) |  |
|  |  |  |  |  |  |  |  |
| **Include all baseline covariates ^b^ (Cox Regression)** | | | | | | | |
| Fully adjusted | 25,598 | 159 | 37,209 | 1.35 (0.89-2.03) | 1.17 (0.69-2.00) | 0.97 (0.41-2.29) |  |
| Weighted | 25,598 | 159 | 37,209 | 1.41 (0.95-2.09) | 1.14 (0.66-1.98) | 0.67 (0.23-1.95) |  |

‘Fully adjusted’ models used multivariable regression, ‘weighted’ models used inverse probability of treatment weighting. Comparisons with small number of outcomes (<5) have been masked. SSRI selective serotonin reuptake inhibitor.

^a^ Subdistribution hazard ratios for competing risk regression.

^b^ Some variables were excluded as the models did not converge. Excluded variables were: AIDs, moderate liver disease, and Huntington’s disease.

**Table S12. Sensitivity analyses for deaths due to diseases of the respiratory system: varying the survival analysis models.**

|  |  |  |  | **Hazard ratios ^a^ (95% confidence interval)** | | |
| --- | --- | --- | --- | --- | --- | --- |
| **Analysis, Model** | **People, No.** | **Deaths, No.** | **Follow-up, years** | **Mirtazapine/**  **SSRI** | **Mirtazapine/ Amitriptyline** | **Mirtazapine/ Venlafaxine** |
| **Main analysis, including results for ‘fully-adjusted’ multivariable regression (Fine-Gray Regression)** | | | | | | |
| Unadjusted | 25,598 | 106 | 37,209 | 2.86 (1.86-4.42) | 1.60 (0.90-2.84) | 2.24 (1.00-5.03) |
| Age-sex adjusted | 25,598 | 106 | 37,209 | 1.24 (0.80-1.94) | 1.21 (0.68-2.14) | 0.73 (0.34-1.55) |
| Fully adjusted | 25,598 | 106 | 37,209 | 1.37 (0.76-2.47) | 1.31 (0.60-2.90) | 0.66 (0.24-1.83) |
| Weighted | 25,598 | 106 | 37,209 | 1.72 (1.07-2.77) | 1.40 (0.73-2.68) | 1.53 (0.62-3.75) |
|  |  |  |  |  |  |  |
| **Complete case analysis** | | | | | | |
| Weighted | 6,794 | 33 | 9,339 | 0.93 (0.36-2.37) | 1.08 (0.34-3.41) | -- |
|  |  |  |  |  |  |  |
| **Include only variables with complete data in models** | | | | | | |
| Weighted | 25,598 | 106 | 37,209 | 1.78 (1.11-2.87) | 1.48 (0.78-2.81) | 1.63 (0.65-4.11) |
|  |  |  |  |  |  |  |
| **Repeat main analysis using Cox regression instead of Fine-Gray regression** | | | | | | |
| Unadjusted | 25,598 | 106 | 37,209 | 2.95 (1.91-4.55) | 1.62 (0.91-2.87) | 2.32 (1.03-5.20) |
| Age-sex adjusted | 25,598 | 106 | 37,209 | 1.30 (0.84-2.01) | 1.27 (0.71-2.26) | 0.75 (0.33-1.69) |
| Fully adjusted | 25,598 | 106 | 37,209 | 1.48 (0.87-2.50) | 1.32 (0.67-2.61) | 0.57 (0.23-1.44) |
| Weighted | 25,598 | 106 | 37,209 | 1.74 (1.08-2.80) | 1.41 (0.74-2.70) | 1.54 (0.63-3.78) |
|  |  |  |  |  |  |  |
| **Including all baseline covariates ^b^ (Cox Regression)** | | | | | | |
| Fully adjusted | 25,598 | 106 | 37,209 | 1.73 (1.01-2.99) | 1.19 (0.59-2.40) | 0.68 (0.26-1.78) |
| Weighted | 25,598 | 106 | 37,209 | 1.88 (1.16-3.05) | 1.58 (0.83-3.02) | 1.60 (0.64-4.03) |

‘Fully adjusted’ models used multivariable regression, ‘weighted’ models used inverse probability of treatment weighting. Comparisons with small number of outcomes (<5) have been masked. SSRI selective serotonin reuptake inhibitor.

^a^ Subdistribution hazard ratios for competing risk regression.

^b^ Some variables were excluded as the models did not converge. Excluded variables were: AIDs, moderate liver disease, and Huntington’s disease.

**Table S13. Sensitivity analyses for deaths due to neoplasms: varying the survival analysis models.**

|  |  |  |  | **Follow-up <2years ^a^**  **Hazard Ratio ^b^ (95% confidence interval)** | | | **Follow-up 2+ years**  **Hazard Ratio ^b^ (95% confidence interval)** | | |
| --- | --- | --- | --- | --- | --- | --- | --- | --- | --- |
| **Analysis, Model** | **People, No.** | **Deaths, No.** | **Follow-up, years** | **Mirtazapine/**  **SSRI** | **Mirtazapine/**  **Amitriptyline** | **Mirtazapine/**  **Venlafaxine** | **Mirtazapine/**  **SSRI** | **Mirtazapine/**  **Amitriptyline** | **Mirtazapine/**  **Venlafaxine** |
| **Main analysis, including results for ‘fully-adjusted’ multivariable regression** | | | | | | |  |  |  |
| Unadjusted | 25,598 | 156 | 37,209 | 3.82 (2.44-5.98) | 0.86 (0.55-1.34) | 2.46 (1.04-5.78) | 2.82 (1.42-5.60) | 9.90 (1.29-75.8) | 3.94 (0.90-17.36) |
| Age-sex adjusted | 25,598 | 156 | 37,209 | 2.08 (1.32-3.29) | 0.74 (0.47-1.16) | 1.19 (0.50-2.84) | 1.44 (0.72-2.88) | 8.21 (1.06-63.4) | 1.74 (0.39-7.78) |
| Fully adjusted | 25,598 | 156 | 37,209 | 1.70 (1.01-2.86) | 1.06 (0.61-1.83) | 1.42 (0.47-4.34) | 1.26 (0.58-2.72) | 7.94 (1.05-59.9) | 1.48 (0.27-8.23) |
| Weighted | 25,598 | 156 | 37,209 | 1.74 (1.06-2.85) | 1.08 (0.64-1.81) | 1.14 (0.42-3.09) | 1.86 (0.89-3.89) | 9.37 (1.20-73.3) | 4.79 (1.00-22.99) |
|  |  |  |  |  |  |  |  |  |  |
| **Complete case analysis** | | | | | | |  |  |  |
| Weighted | 6,794 | 47 | 9,339 | 2.17 (0.85-5.52) | 1.51 (0.63-3.60) | -- | 2.82 (0.76-10.5) | -- | -- |
|  |  |  |  |  |  |  |  |  |  |
| **Include only variables with complete data in models** | | | | | | |  |  |  |
| Weighted | 25,598 | 156 | 37,209 | 1.77 (1.08-2.89) | 1.09 (0.65-1.82) | 1.29 (0.51-3.24) | 1.97 (0.94-4.14) | 9.11 (1.16-71.5) | 5.30 (1.12-25.1) |
|  |  |  |  |  |  |  |  |  |  |
| **Repeat main analysis using Cox regression instead of Fine-Gray regression** | | | | | | | | | |
| Unadjusted | 25,598 | 156 | 37,209 | 3.85 (2.45-6.05) | 0.86 (0.55-1.35) | 2.48 (1.05-5.85) | 2.89 (1.46-5.73) | 10.1 (1.33-76.8) | 4.05 (0.92-17.83) |
| Age-sex adjusted | 25,598 | 156 | 37,209 | 2.10 (1.33-3.31) | 0.74 (0.48-1.16) | 1.20 (0.50-2.83) | 1.47 (0.74-2.91) | 9.00 (1.18-68.5) | 1.72 (0.39-7.62) |
| Fully adjusted | 25,598 | 156 | 37,209 | 1.79 (1.08-2.97) | 1.11 (0.65-1.87) | 1.30 (0.50-3.34) | 1.29 (0.62-2.72) | 8.34 (1.07-64.8) | 1.56 (0.31-7.98) |
| Weighted | 25,598 | 156 | 37,209 | 1.75 (1.07-2.86) | 1.08 (0.64-1.82) | 1.15 (0.42-3.10) | 1.87 (0.89-3.92) | 9.52 (1.22-74.6) | 4.83 (1.00-23.26) |
|  |  |  |  |  |  |  |  |  |  |
| **Include all baseline covariates in models ^c^ (Cox regression)** | | | | | | |  |  |  |
| Fully adjusted | 25,598 | 156 | 37,209 | 1.86 (1.11-3.11) | 1.13 (0.66-1.93) | 1.08 (0.42-2.77) | 1.47 (0.69-3.12) | 9.76 (1.25-76.5) | 1.91 (0.38-9.52) |
| Weighted | 25,598 | 156 | 37,209 | 2.10 (1.25-3.51) | 1.16 (0.66-2.03) | 1.58 (0.61-4.10) | 1.89 (0.89-4.00) | 9.31 (1.19-73.1) | 3.83 (0.79-18.54) |
|  |  |  |  |  |  |  |  |  |  |
| **Exclude people with cancer at baseline (Cox regression)** | | | | | | | |  |  |
| Age-sex adjusted | 24,206 | 66 | 35,397 | 1.64 (0.79-3.42) | 1.08 (0.46-2.49) | 0.80 (0.23-2.80) | 1.17 (0.52-2.67) | -- | -- |
| Fully adjusted | 24,206 | 66 | 35,397 | 1.85 (0.84-4.09) | 1.14 (0.46-2.83) | 0.57 (0.15-2.13) | 1.48 (0.60-3.61) | -- | -- |

‘Fully adjusted’ models used multivariable regression, ‘weighted’ models used inverse probability of treatment weighting. Comparisons with small number of outcomes (<5) have been masked. SSRI selective serotonin reuptake inhibitor.

^a^ All follow-up in the models that did not include the interaction term. ^b^ Subdistribution hazard ratios for competing risk regression. ^c^ Some variables were excluded as the models did not converge. Excluded variables were: AIDs, moderate liver disease, and Huntington’s disease.
